# A critical review of the ecological status of lakes and rivers from Canada's oil sands region

**DOI:** 10.1002/ieam.4524

**Published:** 2021-10-25

**Authors:** Tim J. Arciszewski, Roderick R. O. Hazewinkel, Monique G. Dubé

**Affiliations:** ^1^ Environmental Stewardship Division Alberta Environment and Parks Calgary Alberta Canada; ^2^ Environmental Stewardship Division Alberta Environment and Parks Edmonton Alberta Canada; ^3^ Present address: Cumulative Effects Environmental Inc. Calgary Alberta Canada

**Keywords:** Alberta, Monitoring, Oil sands, Surface water

## Abstract

We synthesize the information available from the peer‐reviewed literature on the ecological status of lakes and rivers in the oil sands region (OSR) of Canada. The majority of the research from the OSR has been performed in or near the minable region and examines the concentrations, flux, or enrichment of contaminants of concern (CoCs). Proximity to oil sands facilities and the beginning of commercial activities tend to be associated with greater estimates of CoCs across studies. Research suggests the higher measurements of CoCs are typically associated with wind‐blown dust, but other sources also contribute. Exploratory analyses further suggest relationships with facility production and fuel use data. Exceedances of environmental quality guidelines for CoCs are also reported in lake sediments, but there are no indications of toxicity including those within the areas of the greatest atmospheric deposition. Instead, primary production has increased in most lakes over time. Spatial differences are observed in streams, but causal relationships with industrial activity are often confounded by substantial natural influences. Despite this, there may be signals associated with site preparation for new mines, potential persistent differences, and a potential effect of petroleum coke used as fuel on some indices of health in fish captured in the Steepbank River. There is also evidence of improvements in the ecological condition of some rivers. Despite the volume of material available, much of the work remains temporally, spatially, or technically isolated. Overcoming the isolation of studies would enhance the utility of information available for the region, but additional recommendations for improving monitoring can be made, such as a shift to site‐specific analyses in streams and further use of industry‐reported data. *Integr Environ Assess Manag* 2022;18:361–387. © 2021 The Authors. *Integrated Environmental Assessment and Management* published by Wiley Periodicals LLC on behalf of Society of Environmental Toxicology & Chemistry (SETAC).

## KEY POINTS

- Contaminants of concern are often found at elevated concentrations adjacent to oil sands mines and some guidelines are occasionally exceeded in lakes and rivers.

- While site preparation and construction activities may influence streams, studies using upstream reference sites to detect change at the downstream exposure locations report differences but often cannot separate natural and anthropogenic causes.

- Paleolimnological reconstructions of biotic commuities from lake cores and contemporaneous collections of invertebrates from lakes do not show evidence of toxicity and instead often suggest increases in primary productivity or good ecological status.

- Preliminary analyses suggest relationships between on‐site industry practices with deposition of contaminants in snow, and with health of fish.

## INTRODUCTION

**Figure 1:**
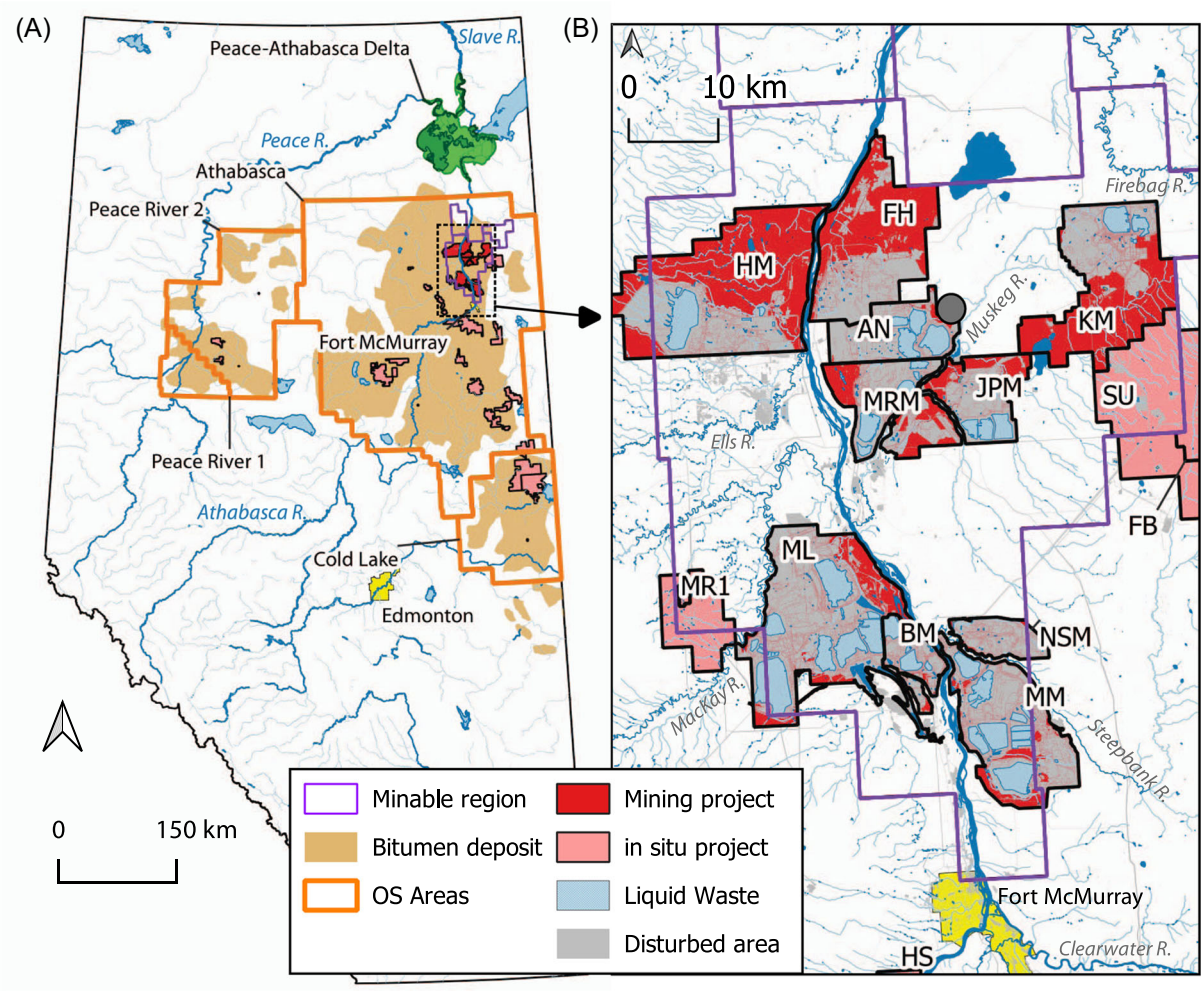
Map of oil sands regions in Alberta, Canada, (A) and minable region (B) showing the bitumen deposit area, oil sands administrative areas (e.g., Athabasca oil sands region), the minable region, boundaries of operating mining and in situ projects (2016), locations of wastewater ponds, and disturbed areas; AN, Syncrude Aurora North; BM, Suncor Basemine; FB, Suncor Firebag; FH, Fort Hills Mine; HM, Horizon Mine; HS, Athabasca Hangingstone; JPM, Jackpine Mine; KM, Kearl Mine; ML, Syncrude Mildred Lake; MM, Suncor Millennium Mine; MR1, Suncor MacKay River; MRM, Muskeg River Mine; NSM, Suncor North Steepbank Mine (also known as Steepbank Mine); and SU, Husky Sunrise. Further landscape and industry details are available in Figure S1 and Table S1; the centroid of the minable region is also shown (grey circle in B)

Oil sands industrial activities (OSIAs) in northeastern Alberta (Figure 1) extract bituminous sands from the McMurray Formation through open‐pit surface mining and in situ recovery. Once extracted, the bitumen is either diluted with lighter hydrocarbons or converted into synthetic crude and transported to other petroleum facilities for further processing (e.g., Giesy et al., 2010; Stringham, 2012). Like other industrial operations, there are known influences on the environment of OSIA. The land is cleared and streams are realigned. Some wastes are also stored on‐site, whereas others are released into the environment. While the permissible activities are governed by facility‐specific Approvals to Operate, the pace of development and the number, scale, and density of individual operations, especially during the 2000s, led to concerns of unanticipated cumulative effects manifesting beyond lease boundaries (Miall, 2013; Schindler, 2010; Schindler, 2013; Table S1). There were also concerns that the existing monitoring was unable to identify these impacts (Miall, 2013; Schindler, 2010; Schindler, 2013). The confidence that the oil sands were being developed responsibly was further eroded and the concerns were amplified after research performed by independent scientists and published in 2009 and 2010 showed an association between contaminant accumulation in snow and proximity to industrial facilities (Kelly et al., 2009, 2010; Miall, 2013).

The erosion of confidence and the amplification of concerns prompted various reactions from the industry, government, and researchers (Miall, 2013). While some novel field studies were initiated quickly (e.g., Frank et al., 2014a; Kurek et al., 2013), reviews of the existing information and the monitoring systems used to inform regulatory decision making requiring more time were also launched (AEMP, 2011; Donahue, 2011; Dowdeswell et al., 2010; Gosselin et al., 2010). The review committees unanimously agreed that the existing monitoring was inadequate and, along with other groups, made many recommendations to improve regulatory oversight in the oil sands region (OSR; e.g., Donahue, 2011; Dowdeswell et al., 2010; Huot & Grant, 2011; James & Vold, 2010; Lott & Jones, 2010; Miall, 2013). Among the recommendations eventually adopted was the formation of a new entity composed of government scientists, third parties, and stakeholders to oversee, coordinate, and conduct integrated regional studies of air, water, and land. First known as the Joint Oil Sands Monitoring (JOSM) program and recently rebranded as the Oil Sands Monitoring Program (OSM; Arciszewski et al., 2021), the work is supported by an annual industry levy (typically ~$50M [CAD]) collected under Alberta's Provincial Oil Sands Environmental Monitoring Regulation (Regulation 226/2013).

Principles to guide the monitoring were also articulated (Dowdeswell et al., 2011). Increasing the scientific credibility of regional monitoring became a priority and was implemented in two primary ways. First, the technical, temporal, and spatial scope of monitoring studies was greatly expanded. Second, peer review of results was required. To meet these (and other) requirements, the frequency of sampling, the number of sites, and the number of measurements were increased (e.g., Glozier et al., 2018), and while some reports have been produced (i.e., Culp et al., 2018), most of the information is now released in peer‐reviewed journals. These approaches greatly increased the amount of information available to assess the state of the ambient environment in the OSR but were also accompanied by a parallel increase in work funded through other sources (Shotyk et al., 2014; Watson et al., 2019).

Although more peer‐reviewed studies are now available, much of the reporting on the environmental performance of the industry and the health of the ambient environment surrounding the facilities, irrespective of funding source, remains isolated to individual and often highly focused, specialized articles (Hopke et al., 2016). An important step in the evolution of regional monitoring is a concerted effort to synthesize and integrate the information available from individual publications in review papers (e.g., Suter, 2013). A review series was launched to synthesize and consolidate the available information aligned with OSM theme areas: air‐, water‐, and land‐, and community‐based monitoring. The purpose of the present work was to identify the state of knowledge, gaps, contradictions, and consensus, and highlight future needs (Suter, 2013) among the relevant peer‐reviewed literature describing the chemical, physical, and ecological status of lakes and rivers in the OSR. In some cases, we also undertook some novel integrated analyses (Arciszewski et al., 2021) using the publicly available or published data and included relevant information from other aquatic and semiaquatic environments, such as snow, bogs, and groundwater. Parallel reviews on air, land, and community‐based monitoring can be found elsewhere (Horb et al., 2021; Roberts et al., 2021b; Beausoleil et al., 2021).

## METHODS

The focus of the present work was on extracting and reviewing information from peer‐reviewed publications examining surface waters. Initial searches for oil sands literature were performed using general oil sands search terms (“oil sand* AND Athabasca OR oil sand* and Alberta” in Scopus and Google Scholar). Initially identified publications were also scoured for additional literature missed in previous searches. Although there are many papers available, we restricted this review to field studies reporting the status of an environmental indicator in the ambient environment. While the primary period of focus was field studies published between 2009 and 2020, additional work published prior to this period was also included where relevant (e.g., Tetreault et al., 2003). One hundred and twenty‐three papers fulfilled these criteria.

Other information was used to contextualize the compiled results and conclusions. While largely beyond the scope of this review, studies relevant for better framing the results of field studies, such as snow toxicity (e.g., Parrott et al., 2018), were also included. Similarly, results in compliance, other regional monitoring, or modeling studies were not a focus of review but were included if deemed relevant (e.g., RAMP, 2016).

Many studies focusing on surface waters in northeastern Alberta have appeared in the peer‐reviewed literature and an organizational scheme was needed to consolidate the information. These studies have been broadly informed by known or conceptual linkages between OSIA and the environment (Wrona et al., 2011), and information on the Pressures, Stressors, Pathways, and Receptors (for definitions see the companion article by Roberts et al., 2021a) discussed either explicitly or implicitly in each manuscript was extracted. This categorization of papers is described in Supporting Information B and was used in additional work (Roberts et al., 2021a). In the current review, the general categorization of papers by conceptual associations was augmented to group, and related information was synthesized by environment type (lakes and streams), physicochemical indicators, exceedances of environmental quality guidelines, and results of biomonitoring. Recommendations for further studies were also made.

While the main focus of this work was on integrated interpretation of the available information, as mentioned previously, we also performed some additional analyses and presentation of data to further explore the implications of papers and any alternative hypotheses or to critically evaluate the results. These additional analyses used data from public sources or data extracted from peer‐reviewed publications. First, the deposition estimates of polyaromatic compounds (PACs) (Manzano et al., 2016) and vanadium (V) in the snow (Gopalapillai et al., 2019) were statistically compared to mine production data (AER, 2021). Median loading of V (mg/m^2^) in the “near field” (≤8 km) was extracted from Gopalapillai et al. (2019). The mean loading of PACs (mg/m^2^) in the source material was not available for an area smaller than 50 km from the Suncor and Syncrude upgrading complexes. To approximate a “near‐field” deposition rate comparable to that of Gopalapillai et al. (2019), the exponential decay functions available from Manzano et al. (2016) were used to estimate the mean deposition of PACs in snow 4 km from Suncor's eastern coke pile; 4 km was selected as the mid‐point of Gopalapillai et al.'s (2019) “near‐field” area.

**Figure 2:**
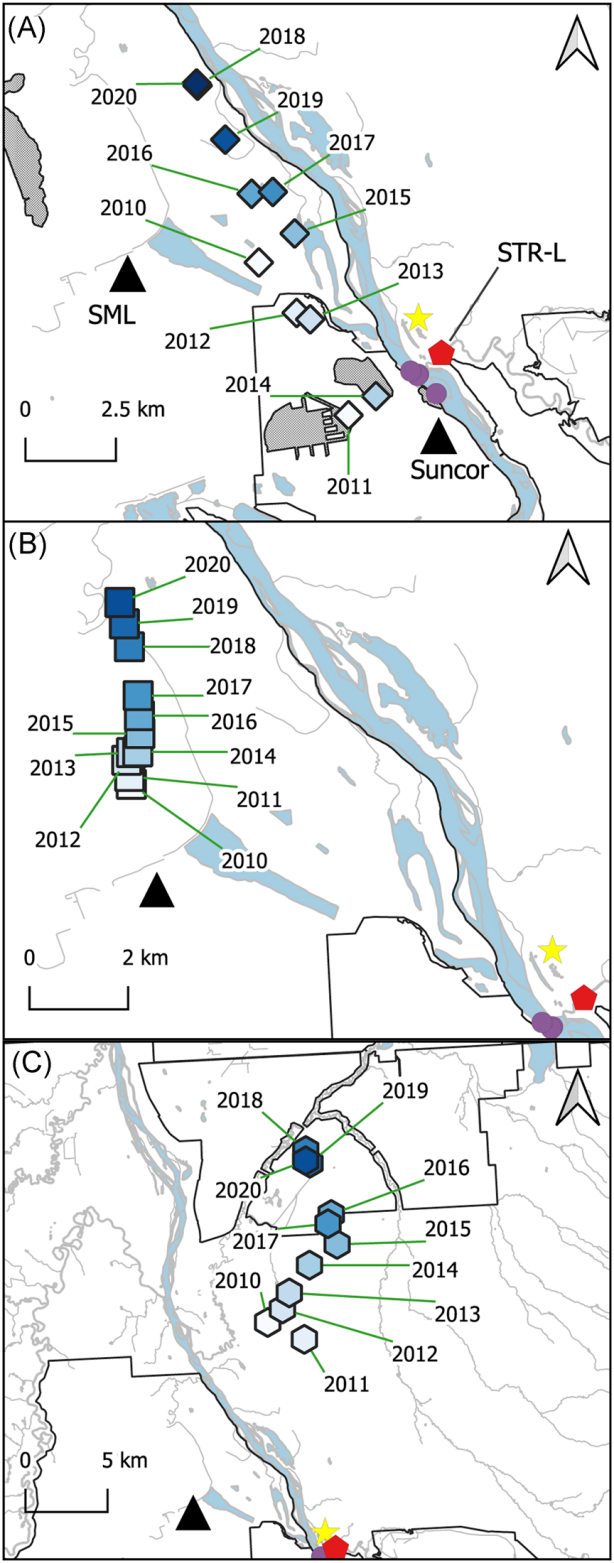
Mean weighted coordinates for (A) annual petroleum coke production, (B) annual closing petcoke inventory, and (C) annual crude bitumen production. The star shows the location of AR6 estimated from Kurek et al. (2013); purple circles show site coordinates of AR6 snow samples available from Manzano et al. (2016). The identity of the mine project areas is shown in Figure 1. SML, Syncrude Mildred Lake fluid coking complex; Suncor, Suncor Basemine delayed coking complex; and STR‐L, fish sampling location shown. The grey shading in Panel A shows areas of petroleum coke storage; the petcoke storage area at Horizon mine is not shown

Second, the means of fish health metrics (gonadosomatic index [GSI], liver‐somatic index (LSI), and condition [K]) for slimy sculpin (*Cottus cognatus*) captured at three locations in the Steepbank River that were obtained from Tetreault et al. (2020) were also compared to some facility‐level data. Slimy sculpin were captured at the lower Steepbank location (STR‐L; Figures 2 and S1) near the mouth and adjacent to the Suncor Basemine and the Steepnak and Millenium Mines. The middle Steepbank location (STR‐M; Figure S1) is upstream of some, but not all, development in the Steepbank basin. The third sampling location, upper Steepbank (STR‐U; Figure S1), is upstream of the mining development in the Steepbank River. These analyses used fish collected from 2010 to 2013 to match with the facility data available at the time of preparation of this article.

The mine production data were obtained from records submitted by companies to the Alberta Energy Regulator and compiled in the annual Statistical Report 39 (ST39; AER, 2021). Eight industrial descriptors (petroleum coke as fuel, petroleum coke production, closing inventory of petroleum coke in March, synthetic crude production, crude bitumen production, process gas as fuel, natural gas as fuel, and synthetic crude as fuel) from both Syncrude Mildred Lake and Suncor Basemine were obtained for these exploratory analyses. All industrial metrics, except the closing inventory of petroleum coke in March, were calculated as the sum (e.g., December to March) of the relevant winter season. For brevity, and given the patterns observed in snow and the proximity of the STR‐L site to Suncor's coker, the fish metrics were only compared to petroleum coke listed as fuel from June to September of 2010–2013 at the Suncor Basemine.

Both the snow and fish analyses were performed with a linear model (“lm” function in R) and log_10_‐transformed data. Residual diagnostics indicated no violations of the assumptions of a linear model, although this was challenging to gauge with small sample sizes.

Additional analyses were also performed. The pH of snow samples from 2014 was used to interpolate spatial patterns using Inverse Distance Weighting (distance coefficient = 2) in QGIS 3.16 (https://qgis.org). We also used production and consumption data to perform spatial analyses, including production‐weighted mean coordinates (“Mean coordinates” function in QGIS 3.16) to evaluate descriptors, such as the “center of mining” (and its variants) with the georeferenced locations collectively known as AR6 (e.g., Kelly et al., 2009; Manzano et al., 2016). The mean coordinates weighted by (1) annual petcoke production, (2) closing annual inventory of petroleum coke, and (3) crude bitumen production at mines from 2010 to 2020 were calculated using the centroids of mining leases. The centroid of the minable region was also calculated (Figure 1).

Some other data were also extracted from individual manuscripts or from public databases and reproduced here to enhance understanding. This included polyaromatic hydrocarbons (PAHs) in lake and stream sediments and compared to sediment quality guidelines. Concentrations of metals in lake sediments, the total annual area of Alberta burned by wildfires, cumulative land disturbances per watershed, and reconstructions of chlorophyll *a* are also shown. Fluoranthene and pyrene were also obtained from multiple studies examining lake sediments (e.g., Evans et al., 2016), from papers reporting chemical profiles of source materials (e.g., Landis, Studabaker, et al., 2019), and measurements (“source testing” estimation method, “stack/point” category, and “releases to air” group) reported in Canada's National Pollutant Release Inventory (NPRI) for Syncrude Mildred Lake (NPRI ID# 2274) and Suncor Basemine (NPRI ID# 2230). These data were used to calculate a diagnostic ratio of fluoranthene to fluoranthene and pyrene (e.g., Yunker et al., 2002) commonly used in the region to explore patterns within and among studies.

## LITERATURE REVIEW

### Status of lakes

#### Physicochemical status of lakes

##### PACs and elements

A primary focus of many studies in the OSR has been the measurement of CoCs in the sediments of lakes in, or adjacent to, the minable region north of Fort McMurray (Figures 1 and S1). Among the many lakes evaluated across the region, the greatest influence of OSIA, including increases in PACs appears in two lakes, NE13 and NE20, adjacent to the first two mines (Suncor Basemine and Syncrude Mildred Lake; Figures 1 and S1, Table S1; Ahad et al., 2015; Evans et al., 2016; Jautzy et al., 2013; Kurek et al., 2013; Summers et al., 2016, 2017). Together with results from snow, lichens, and peat moss, these data also show a nonuniform deposition of CoCs within ~50 km of the Syncrude and Suncor upgrading complexes, supporting the regional influence of these sources (Chibwe et al., 2019; Cho et al., 2014; Kelly et al., 2009, 2010; Landis, Studabaker, et al., 2019; Manzano et al., 2016; Y. Zhang et al., 2016). However, the influence of other facilities is also likely within this 50 km zone (Sharkbite and Kearl; Figure S2; Evans et al., 2016) and in lakes on the eastern margins of the minable region adjacent to in situ facilities (e.g., L185, P7, and E15; Figure S3). Researchers also suggest that there is evidence of OSIA‐derived PACs in lakes in the PAD (Chibwe et al., 2019; Jautzy, Ahad, Gobeil, et al., 2015; Manzano et al., 2017), the Cold Lake region (Korosi et al., 2013, 2016), Namur Lake in the west (90 km northwest of Suncor's Basemine; Kurek et al., 2013), and, while subtle, potentially in lakes in northern Saskatchewan and eastern Alberta (up to 85 km northeast and east of Suncor's Basemine (Ahad et al., 2015; Jautzy et al., 2013). While the timing of changes in indicators suggests the influence of early and/or local operation of facilities, earlier effects potentially associated with technology development may also be apparent (Cooke et al., 2017; Skierszkan et al., 2013).

The elevated concentrations, flux, or enrichment of anthropogenic CoCs in local lakes, while nuanced and affected by multiple sources, such as forest fires (e.g., Ahad et al., 2015) and potentially urbanization (Figure S3), have largely been attributed to OSIA via raw bitumen and petroleum coke dust (e.g., Zhang et al., 2016) and upgrader emissions (e.g., Kelly et al., 2009). Other sources of fugitive dust, such as mining roads, mine pits, overburden piles, and tailings pond dykes, may also influence the observed patterns (Cooke et al., 2017; Gopalapillai et al., 2019; Landis et al., 2012; Shotyk et al., 2014). These data and conclusions, along with others (Korosi et al., 2016; Munkittrick & Arciszewski, 2017; Skierszkan et al., 2013; Summers et al., 2017), also suggest a greater effect of mining on the ambient environment than in situ.

While many measurements of PACs suggest increases in concentrations or deposition rates (e.g., Chibwe et al., 2019; Kurek et al., 2013), declines have also been documented among some estimates of industrial influence. The relative flux and enrichment factors of N‐ and S‐PACs have declined in lake NE20 since in the most recent sediment core section (~2015; Chibwe et al., 2020). Data extracted from Manzano et al. (2016) for the high‐deposition zone used by Gopalapillai et al. (2019) suggest that PAC deposition 4 km from the Suncor and Syncrude upgraders decreased from 2011 to 2014, paralleling results from other reanalyses of PAC deposition in snow using the 2012–2014 data (McNaughton et al., 2019). Deposition rates of metals in snow and lake sediments at sites close to Syncrude Mildred Lake and Suncor Basemine have also been declining since at least the 1970s and may be associated with the installation of electrostatic precipitators at Suncor, potentially controlling high‐vanadium fly ash of combusted delayed coke (Bryers, 1995) or other general improvements in the environmental performance of facilities (Chibwe et al., 2020; Cooke et al., 2017; Gopalapillai et al., 2019). Discrepancies between elemental measurements and PACs, including increases in PACs in the sediments of local lakes over time (e.g., Chibwe et al., 2020; Kurek et al., 2013) potentially suggest that different mechanisms may be influencing the occurrence of materials derived from industry appearing in various media.

Other patterns have also emerged from studies of lake sediments and snow. Elemental concentrations in lake sediments also suggest an influence of local geology on the chemistry of lakes (Cooke et al., 2017). However, as seen in lake sediment Cd, the analytical imprecision of samples collected in 2014 may limit the interpretation of these data (Figure S5). Other researchers suggest that natural geological features may not influence the patterns of PAHs in lake sediments (Kurek et al., 2013), whereas others suggest that there may be a role of groundwater seeps (Evans et al., 2016).

The influence of globally circulating CoCs was also apparent in some studies. Reduced inputs of Pb and Hg originating from global sources and observed elsewhere (e.g., Rauch & Pacyna, 2009; Selin, 2009) have been identified in the OSR (Cooke et al., 2017; Shotyk et al., 2016). There may be an influence of globally circulated V (Hope, 2008; Rauch & Pacyna, 2009; Schlesinger et al., 2017) in some lakes, as suggested by an association between concentrations of Pb and V from Kearl Lake (e.g., Figure S4), NE13, NE20, and lakes grouped by distances from Syncrude Mildred Lake and Suncor Basemine, but these observed patterns are more likely attibutable to either mineral erosion in the watershed, inputs from local anthropogenic sources, or both (Cooke et al., 2017). However, deposition from global sources may also be apparent in Cd from the minable region lakes, but as mentioned already, insufficient resolution of measurements in 2014 obscures the potential patterns in lakes sampled that year (Figure S5). Declines in the measurements of some metals over time, suggesting a role of deposition of substances originating from global sources, have also been observed in lakes from the PAD (Wiklund et al., 2012) and the Cold Lake Region (Skierszkan et al., 2013).

##### Describing spatial patterns of CoC deposition in the OSR

Describing the concentrations and spatial deposition of CoCs in the OSR is common in the peer‐reviewed literature and some typical approaches have emerged. Most studies use the AR6 location as a proxy for industrial activity and distance to this point is often used to describe areas affected by elevated concentrations of CoCs originating from OSIA (Ahad et al., 2015; Brunet et al., 2020; Chibwe et al., 2019; Emmerton et al., 2018; Gopalapillai et al., 2019; Jautzy et al., 2013; Kelly et al., 2009, 2010; Laird et al., 2013; Neville et al., 2014). While the AR6 location was originally described as a halfway point between the Syncrude and Suncor upgraders to reflect emissions from these sources (Kelly et al., 2009), the location has also been used as a proxy for the “main mining center” (Ahad et al., 2015; Jautzy et al., 2013), the “center of the main development area” (Summers et al., 2016), and the “center of open‐pit mining activities” (Cooke et al., 2017). While these descriptors used to describe the periods in which Suncor Basemine and Syncrude Mildred Lake were the only operating commercial facilities may be accurate, some of these latter descriptors applied to later periods, such as after 2000 (and the expansion of the industry into the Muskeg basin, for example), may not.

We evaluated the correspondence between these written descriptions of AR6 and potential numerical equivalents using facility‐level production data available at the time this manuscript was prepared (2010–2020). When the general descriptions of AR6 are compared to some of the possible quantitative analogs, such as mean coordinates weighted by crude bitumen production, stockpiled petroleum coke, or the centroid of the minable region, only the mean coordinates weighted by annual petcoke production in 2011–2014 roughly correspond to the AR6 location described by Kelly et al. (2009; Figures 1 and 2). The mean coordinates calculated here suggest that the broad descriptions of AR6 as the center of mining are not accurate in all its applications. These mean coordinates further suggest that rather than as a regional center of the influence of open‐pit mining, AR6 may be better described as the center of regional petroleum coke production or as a surrogate of petcoke storage at Suncor Basemine (Figure 2). However, the mean coordinates also suggest that this description is limited to data collected from 2011 to 2014 and may also explain some discrepancies in the peer‐reviewed literature when the AR6 coordinate is used to orient spatial gradients (Gopalapillai et al., 2019; Manzano et al., 2016; Summers et al., 2016). Our analyses further suggest, similar to other work already carried out (e.g., Gopalapillai et al., 2019; Kirk et al., 2014; Landis, Studabaker, et al., 2019; Landis, Berryman, et al., 2019; Manzano et al., 2016; Willis et al., 2018), that shifting to geospatial approaches better suited for the monitoring objectives may be necessary. The analyses further suggest that the use of spatial coordinates to describe the configuration of facilities on the landscape be more deliberate (e.g., Gopalapillai et al., 2019; Landis, Studabaker, et al., 2019).

##### Identifying industrial sources using facility‐level fuel and production data

Researchers suggest that Suncor's coke stockpiles are a potent source of some CoCs found in various media beyond the lease boundaries, such as PACs (e.g., Chibwe et al., 2019). However, Suncor's main stack is also present in the vicinity of Suncor's eastern‐most coke pile and samples of snow used to approximate AR6 (Figure 2). While a portion of petroleum coke is combusted as a typical part of the fluid coking process used at Syncrude (Speight, 2016), it is also used to fuel separate boilers at the Suncor Basemine (McNaughton et al., 2019), creating a potential confounding effect when studies examine potential sources. Previous researchers have alluded to the potential utility of facility‐specific data in explaining the occurrence of CoCs in the ambient environment (e.g., Gopalapillai et al., 2019) and exploratory analyses using these data were performed here.

**Figure 3:**
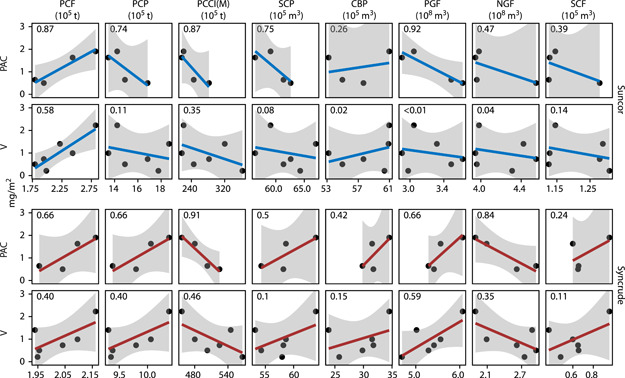
Comparisons of the estimated average loading of PACs (2011–2014; Manzano et al., 2016) and V (2011–2016; Gopalapillai et al., 2019) in snow within 8 km of AR6 to winter (December–March) production and fuel use data at the Suncor Basemine and Syncrude Mildred Lake. CBP, crude bitumen production; NGF, natural gas as fuel; PCCI(M), petroleum coke closing inventory in March; PCF, petroleum coke as fuel; PCP, petroleum coke production; PGF, process gas as fuel; SCP, synthetic crude production; and SCF, synthetic crude as fuel. Production and fuel data were obtained from ST39 reports published by the Alberta Energy Regulator (AER, 2021); values in the upper left of each sub‐pane are the *R* ^2^ values for log‐transformed linear models. Shading represents the 95% confidence interval of each regression line

Among the industrial data available in the AER ST39 reports, such as the fuel use of petroleum coke compared to estimated deposition of PACs and V in snow from the near‐field area defined in Gopalapillai et al. (2019), none were statistically significant at the 0.2% level (Bonferroni adjustment for 32 comparisons; *p *≥ 0.038; Figure 3). However, the mass of petcoke categorized as fuel at Suncor each winter explained 87% and 58% of the variability of the mean PAC (2011–2014) and the median V (2011–2016) deposited in snow, respectively (Figure 3). While weaker, the same pattern was observed using these data from Syncrude (*R* ^2^ = 0.66 and 0.40 for PACs and V, respectively). Process gas as fuel at Suncor also suggests a negative influence on PACs (*R* ^2^ = 0.92), but a positive influence was observed at Syncrude (*R* ^2^ = 0.66). While the relationship of PAC deposition in snow and petroleum coke production at Suncor was negative (*R* ^2^ = 0.74), this relationship was positive at Syncrude, also with an *R* ^2^ = 0.66; the similarities of the *R* ^2^ among petroleum coke as fuel, petroleum coke production, and process gas as fuel at Syncrude match the process relationships of these substances at that facility, but also reflect differences in fluid and delayed coking technologies (Speight, 2016). The similarity of these *R* ^2^ at Syncrude also highlights these industry‐reported values as potentially complex indices capturing many on‐site phenomena.

Other patterns also emerge from these comparisons. In contrast to petroleum coke as fuel, the closing inventory of petcoke at the end of each March in each respective study year at both facilities was negatively related to PAC deposition in snow (*R* ^2^ ≥ 0.87; Figure 3). Similarly, greater volumes of NGF at both sites (*R* ^2^ = 0.47 and *R* ^2^ = 0.84, respectively) reduce the deposition of PACs in snow, but only in V at Syncrude (*R* ^2^ = 0.35); the volume of NGF at Suncor has no substantive influence on V deposition in the snow (*R* ^2^ = 0.04). Differences in *R* ^2^ for both PACs and V compared to NGF are likely associated with how this substance is used at these two facilities. As suggested by other research (e.g., Chibwe et al., 2019; Gopalapillai et al., 2019), we observed an overall greater influence of production and fuel use from both facilities on PACs compared to V.

While there are potential limitations of these analyses, including sample sizes, the heteroskedasticity of residuals near AR6 (Manzano et al., 2016; Figure S6), potential collinearity among industrial descriptors, and point clustering (e.g., NGF at Suncor compared to PAC deposition; Figure 3), these data further suggest that the use of petcoke as fuel at the Suncor Basemine and process gas as fuel at Syncrude likely contributes to patterns reported in the literature largely, but not solely, attributed to wind‐blown dust (Ahad et al., 2015; Chibwe et al., 2019; Gopalapillai et al., 2019; Jautzy, Ahad, Gobeil, et al., 2015; Kelly et al., 2009; Landis et al., 2012; Manzano et al., 2017; McNaughton et al., 2019; Shotyk et al., 2016; Xing et al., 2020; Xu, 2018; Yi et al., 2015). Relationships between PAC and V deposition with crude bitumen and synthetic crude production at Syncrude also support hypotheses of both fugitive dust‐ and process‐related emissions.

The results suggesting the roles of fuels may, more specifically, also explain apparent discrepancies in the literature. These discrepancies include declines in N‐ and S‐PACs in sediments of NE20 since ~2015 (Chibwe et al., 2020) and less deposition of metals in snow decades after the installation of electrostatic precipitators and transition to truck and shovel mining (Cooke et al., 2017; Gopalapillai et al., 2019). Differences in Hg loading in the snow between 2011 and 2015 (Kirk et al., 2014; Willis et al., 2018), PAH concentrations and accumulation patterns between NE20 and NE13 (Kurek et al., 2013), and discrepancies in source–receptor profiling (Chibwe et al., 2019; Jautzy, Ahad, Gobeil, et al., 2015; Manzano et al., 2017) may also be explained by industrial fuels and production practices over time and among facilities. While the differences could also be related to the choice of statistical analysis or the mass of stored petcoke, uses of petroleum coke on‐site beyond storage may also explain the discrepancies in PAC deposition near the Horizon mine among studies (Landis, Studabaker, et al., 2019; Manzano et al., 2016; McNaughton et al., 2019). The roles of different on‐site sources may also explain differences in the deposition of CoCs, such as V and PACs, in the sediments of the NE13 and NE20 Lakes (Cooke et al., 2017; Kurek et al., 2013). These data suggest that the inclusion of estimates of intensity to facility occurrence may be powerful additions to studies examining the influence of industrial activity (Gopalapillai et al., 2019; Landis, Studabaker, et al., 2019). For example, the facility‐level data may also account for maintenance or other unplanned shutdowns that may influence observations in environmental indicators (e.g., Horizon reported no production from February to July of 2011; AER, 2021).

Further analyses may be warranted, but may need to reconcile potential nuances. These nuances include facility‐specific activities (e.g., Gary et al., 2007; Speight, 2016), details of items listed in annual statistical reports released by the Alberta Energy Regulator, and the role of ambient temperature in emission and eventual deposition of materials in snow versus other media during other seasons. Results from the peer‐reviewed literature also warrant deeper consideration, including the role of wind‐blown petcoke (Zhang et al., 2016), increases in parent PAHs, alkylated‐PACs, and dibenzothiophenes in NE20 (Chibwe et al., 2020) and some other local lakes (Kurek et al., 2013), patterns suggesting local and regional sources (Evans et al., 2016; Figure S2), and potential roles of forest fires or other sources (e.g., Ahad et al., 2015).

Most importantly, these preliminary results suggest associations between specific industrial practices at specific facilities (Figure S7) and the environmental health of surrounding areas. These hypothesized relationships are also testable. Suncor is in the process of decommissioning its coke‐fueled boilers with natural gas cogeneration plants (Suncor, 2021). This process upgrade is scheduled for completion in 2024 and can be used to further evaluate the importance of potential sources using a before–after‐control‐impact (BACI) design. However, the need to further investigate these data or engage in additional studies within a regulatory monitoring program will also depend on other criteria, including evidence suggesting that productivity is increasing in local lakes, including NE13 and NE20 (Kurek et al., 2013; Summers et al., 2016).

##### PAH ratios

A significant challenge in the oil sands literature, and monitoring in the region, is the lack of necessarily relevant and standard criteria for evaluating results. For example, many ratios of PAHs are commonly used (Ahad et al., 2015; Jautzy et al., 2013; Kurek et al., 2013; Thienpont et al., 2017) and some, such as the ratio of fluoranthene to fluoranthene and pyrene (FL/(FL + PY)), are specifically recommended (Evans et al., 2016). However, various thresholds to identify the potential origin of PAHs have appeared in the peer‐reviewed oil sands literature. Among estimates of the FL/(FL + PY) ratio available in the oil sands literature, Ahad et al. (2015), Evans et al. (2016), and Birks et al. (2017) used two thresholds to identify three possible groups of sources: <0.4 suggesting petrogenic sources, 0.4 ≤ *x *≤ 0.5 suggesting petroleum combustion sources, and >0.5 suggesting pyrogenic sources (De La Torre‐Roche et al., 2009; Tobiszewski & Namieśnik, 2012; Yunker et al., 2002). In contrast, Jautzy et al. (2013) used specific estimates of materials from Yunker et al. (2002) to define petrogenic and pyrogenic ranges. Others have also distinguished pyrogenic and petrogenic sources using 0.4 (Thienpont et al., 2017) and 0.5 (Kurek et al., 2013) and did not use the 0.4–0.5 petroleum combustion range. These different ranges can create interpretative challenges if the categorization is retained without its numerical description.

Notwithstanding other inherent challenges with identifying sources using PAH ratios (Galarneau, 2008), an important implication also arises from the different thresholds used to evaluate ratios and identify relevant patterns. More specifically, applying the “petroleum combustion” range for the FL/(FL + PY) ratio (0.4–0.5) to the results of Kurek et al. (2013) may change the interpretation of the results from Namur Lake. At Namur Lake, the ratio of FL/(FL + PY) in sediments was (on average) ~0.5 before the initiation of OSIA and ~0.48 afterward (Kurek et al., 2013), which is consistent with the influence of a novel petrogenic source. While not definitive, a value of 0.48 of the FL/(FL + PY) ratio after the onset of oil sands development creates an interpretative challenge. This value falls within the “petrogenic combustion” range and is consistent with a regional combustion signal (McNaughton et al., 2019). However, a recreational fishing lodge accompanied by vessels with outboard motors also operates from the shores of Namur Lake (Hazewinkel et al., 2008), which may also be a source of PAHs to the lake and its sediments.

**Figure 4:**
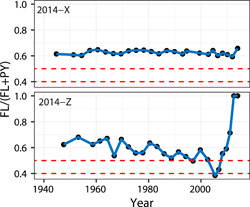
Ratio of fluoranthene to fluoranthene and pyrene (FL/(FL + PY)) in sediment cores collected from two lakes (2014‐X and 2014‐Z) adjacent to Namur Lake compared to estimated year; locations are shown in Figure S1. Horizontal dashed lines indicate ranges for suggesting the average origin of PAHs using the FL/(FL + PY) ratio (pyrogenic, petrogenic, and petroleum combustion; see Yunker et al., 2002); data are available from https://www.canada.ca/en/environment-climate-change/services/oil-sands-monitoring/monitoring-water-quality-alberta-oil-sands.html. Core section dates are available from Summers et al. (2016)

While the true source of the change in the FL/(FL + PY) ratio in sediments of Namur Lake is not currently clear, other data are available to examine this question. Sediment cores at two additional lakes within 5 km of Namur Lake (2014‐X and 2014‐Z; Figures 4 and S1) have also been collected (Summers et al., 2016). The mean values of the FL/(FL + PY) ratios in 2014‐X and 2014‐Z are, respectively, 0.62 and 0.63 before 1967 and 0.63 and 0.6 afterward, suggesting no substantive change associated with the beginning of large‐scale commercial oil sands development. However, summary statistics, such as means, can obscure directional temporal patterns based on individual (although, in this case, not independent) data points even when estimates of variability are provided. Among individual core sections from these two lakes, the FL/(FL + PY) ratio in the sediments of Lake 2014‐X is consistently in the “pyrogenic range” (Figure 4). However, there is evidence of a temporal drift downward of the FL/(FL + PY) ratio in sediments of lake 2014‐Z (and 0.49 in the later 1990s) until ~2000, followed by estimation of ratios of 0.38 and 0.43 between 2005 and 2007 (Figure 4). After this, the FL/(FL + PY) ratio climbs to, and, reaches 1 (Figure 4). While the FL/(FL + PY) ratio equaling 1 is related to the analytical imprecision of pyrene, the FL/(FL + PY) ratios of surface sediments from Namur Lake collected in 2014 and 2015 were 0.48 and 0.38, respectively (RAMP, 2016) supporting the earlier conclusion of Kurek et al. (2013). These data and others from additional lakes, such as P13 and L60 (Figures S1 and S2), suggest that a more rigorous study may be needed to reconcile the conflicting evidence of the sources of PACs in the sediments of Namur Lake. However, similar analyses may also be necessary at other locations showing conflicting evidence; surficial sediments and two lake cores from Kearl (also called RAMP 418 and Kearle) show differing patterns and, among the two collected cores, slightly different interpretations based on the conventional thresholds in the FL/(FL + PY) ratio between 2000 and 2012 (Figure S8).

Other potential challenges with the use of pre‐determined and generic criteria from the literature for identifying the influence of industrial activity in the OSR can be demonstrated using the FL/(FL + PY) diagnostic PAH ratio. Among some study lakes (but not all), there is evidence of historical petrogenic influence predating the onset of commercial industrial activities in 1967 coupled with (also not in all cases) subtle changes afterward (Ahad et al., 2015; Evans et al., 2016; Jautzy et al., 2013; Jautzy, Ahad, Gobeil, et al., 2015; Jautzy, Ahad, Hall, et al., 2015; Korosi et al., 2013; Kurek et al., 2013; Summers et al., 2016; Figure S2). The observed petrogenic PAHs in some lake sediments before ~1967 could be natural, but could also be associated with historical exploration activities in the 1950s, construction of the Great Canadian Oil Sands plant (1964–1967), early activities at Bitumont in the 1920s–1940s, or process development at Abasands in the 1930s (e.g., Akena, 1979; Smith, 1981; Tingley & Parker, 1980). While some of these patterns may also be affected by transport kinetics and differential scavenging (e.g., Zhang, Cheng, et al., 2015), the patterns in lakes, such as ALE (Figure S2) are, however, in stark contrast to changes observed in some lakes within the minable region and around its periphery, including abrupt shifts and/or gradual changes potentially associated with optimization of facility performance during the following commercial operation (Figure S3). In sum, the FL/(FL + PY) ratio in these two groups of lakes, those outside and within the minable region (Figures S2‐S3), suggest that preindustrial baselines and expected ranges may not always be easily defined using specific dates, such as 1967 or with conventional threshold values for ratios. However, they often might be. The results also suggest the potential need for site‐specific criteria to identify the onset and evolution of industrial influence in some cases, but determining when these additional criteria are needed may be challenging.

In addition to site‐specific criteria, other approaches may overcome some of the interpretative challenges. For example, using multiple ratios may also resolve some of these issues of identifying industrial influence (e.g., Jautzy et al., 2013). While other ratios may also be subject to similar challenges as those already described for the FL/(FL + PY) ratio, they can also create other challenges. Detection limits for some PAHs may not be sufficient to calculate ratios, multiple ratios may provide conflicting evidence, and some, such as alkylated PACs, needed for some ratios (e.g., Evans et al., 2016), may not be measured routinely. Alternative chemical analyses may also resolve some of these issues, but they can be technically challenging to perform (i.e., Jautzy, Ahad, Hall, et al., 2015). While the criteria for pursuing more sophisticated analytical chemistry within the portion of regional monitoring associated with regulatory requirements of facilities in the OSR have not been identified, conflicts such as those described above may suffice.

##### Source profiles

There are additional factors that may affect the success of any future analyses using PACs. Within the published literature, chemical profiles of various materials are commonly used to identify likely sources (Chibwe et al., 2019; Landis, Studabaker, et al., 2019; Manzano et al., 2017). However, when compiled, there is variability within types, including the PAC profiles of various materials including upgrader emissions, petroleum coke, and oil sand (Harner et al., 2018; Jariyasopit et al., 2018; Landis, Studabaker, et al., 2019; Yang et al., 2011; Table 1). Among various samples, the FL/(FL + PY) ratio, chosen as a straightforward example, ranges from 0.031 to 0.284 in oil sands and falls within the petrogenic range. In contrast, the FL/(FL + PY) ratio ranges from 0.189 to 0.261 in delayed petcoke, from 0.184 to 0.462 in fluid petcoke, and from 0.029 to 0.165 when the type of petcoke is not specified. While many of these data also fall into the petrogenic range of this ratio, they also suggest potential challenges in identifying the FL/(FL + PY) petroleum combustion range if fluid petcoke is present in the environmental samples.

**Table 1:** Diversity of fluoranthene/(fluoranthene plus pyrene) (FL/(FL + PY)) ratios among petroleum coke, raw oil sand, and stack emissions obtained from the peer‐reviewed literature and the NPRI (National Pollutant Release Inventory: https://open.canada.ca/data/en/dataset/40e01423-7728-429c-ac9d-2954385ccdfb); NPRI stack emissions (“source testing” for “stack/point” and “releases to air”) for Suncor Basemine (SBM) and Syncrude Mildred Lake are median (range)

Source/material	Details	$\frac{\mathrm{FL}}{\mathrm{FL}+\mathrm{PY}}$	Data source	Notes
Petroleum coke	Delayed coke	0.253	Zhang et al. (2016)	
		0.261		
		0.189	Jariyasopit et al. (2018)	*n* = 3*
	Fluid coke	0.462	Zhang et al. (2016)	
		0.232		
		0.184	Jariyasopit et al. (2018)	*n* = 2*
	Not specified	0.029	Landis, Studabaker, et al. (2019)	
		0.165	Harner et al. (2018)	
Oil sand	AOS1	0.245	Yang et al. (2011)	
	AOS2	0.227		
	AOS3	0.229		
	OS Ore	0.031	Zhang et al. (2016)	
	OS Ore	0.284	Jariyasopit et al. (2018)	*n* = 3*
	Raw Oil Sand	0.087	Landis, Studabaker, et al. (2019)	
	Weathered OS	0.091	Zhang et al. (2016)	
Stack emissions	Upgrader	0.235	Harner et al. (2018)	
		0.774	Landis, Studabaker, et al. (2019)	
	SBM	0.579 (0.509–0.595)	NPRI	2003, 2004, 2009–2019
	SML	0.381 (0.301–0.390)		2003–2010

*Note*: Values marked with asterisks (*) are reported in source material as averages.

Other sources also show discrepancies among the commonly applied thresholds for the FL/(FL + PY) ratio. Among the “upgrader emissions,” only two estimates of the FL/(FL + PY) ratio were found in a scan of the literature: 0.235 and 0.774. A slightly narrower range of 0.301–0.595 was obtained from measured stack emissions from the Syncrude Mildred Lake Mine and the Suncor Basemine reported in the NPRI. While the FL/(FL + PY) ratio from these individual facilities falls into petrogenic (Syncrude: 0.301–0.390) and pyrogenic (Suncor: 0.509–0.595) ranges, the generic descriptors of upgrader and/or stack emissions available from the literature and from pooling the NPRI data from Syncrude and Suncor span the petrogenic, petroleum combustion, and pyrogenic threshold values. While some of the source variability, especially from stacks, may differ both by the facility, with the specific composition of fuels being combusted, and with operational alterations (Speight, 2016; Wang et al., 2012), these patterns suggest a potential and significant challenge in analyses using chemical profiles of substances and the representativeness of the source samples (Chibwe et al., 2019; Jautzy, Ahad, Gobeil, et al., 2015; Landis, Studabaker, et al., 2019; Manzano et al., 2017). While there may also be potential challenges when all relevant sources are not included in a study (Chibwe et al., 2019; Manzano et al., 2017), these data suggest that a complete (and growing) library of materials may be required to capture the variability of individual sources. Similar to other potential analyses to resolve challenges of chemical profiling, the criteria for initiating this work within a regulatory monitoring program have not been identified, but the discrepancies outlined here may be sufficient.

##### Acidification

Studies of lakes in the OSR have also focused on the potential threat of acidification via the emissions of N and S into the atmosphere. While an effect is hypothesized (Anas et al., 2014; Cathcart et al., 2016; Makar et al., 2018; Scott et al., 2010), most studies show no discernible responses consistent with the alteration of the pH of lakes over time (i.e., Anas et al., 2014; Hazewinkel et al., 2008; Laird et al., 2013, 2017). Some evidence has been found in two lakes east of the minable region (~54 and ~80 km west of Suncor's coker, respectively [Curtis et al., 2010; Laird et al., 2013]), but these changes have not been specifically linked with OSIA. In contrast, alkalization of lakes has been more commonly observed (Curtis et al., 2010), and researchers have suggested that lake characteristics (similar to conclusions regarding Hg [e.g., Emmerton et al., 2018]) may be the driving factors in changes in pH (Curtis et al., 2010). Other work suggests a role of fugitive dust rich in cations (Fenn et al., 2015), but analyses of lake sediments have shown no discernible change in Ca over time (Cooke et al., 2017). These data potentially suggest the overlapping but also unique zones of influence of different OSIA‐derived materials and interactions with natural factors, such as assimilative capacity, and reinforce patterns already suggested in measurements of PACs and metals in snow and other media (Cooke et al., 2017; Kelly et al., 2010).

#### Exceedances of environmental quality guidelines in lakes

Assessments of the potential risks of CoCs to organisms residing in lakes have been carried out using environmental quality guidelines. Among PAHs in sediments, the interim sediment quality guidelines (ISQG) were not exceeded in PAD23 (Jautzy, Ahad, Gobeil, et al., 2015), two lakes east of the minable region (Jautzy et al., 2013), two lakes from the Cold Lake area (Korosi et al., 2013), and in five of six study lakes north of Fort McMurray, including lake NE13 (Kurek et al., 2013). In the sixth lake, NE20, sediment guidelines for phenanthrene, pyrene, benz*(a)*anthracene, chrysene, benzo*(a)*pyrene, dibenz*(a,h)*anthracene, and 2‐methylnaphthalene were exceeded (Kurek et al., 2013), and the exceedances have become more frequent since the 1980s; the location of NE20 and the timing of increases suggest a potential association with operations at Syncrude Mildred Lake, but may also be associated with greater activity in the Muskeg basin. Shipyard Lake is within the high‐deposition zones defined in other studies (i.e., Manzano et al., 2016) south of Suncor's upgrading complex (Figure S1). Exceedances of PAH ISQGs were also commonly observed in surface sediments collected at this location (Evans et al., 2016) similar to NE20, but in contrast to NE13 (Kurek et al., 2013). There may, however, be potential challenges with different results from coring compared to surficial sediments or of combining results from multiple studies (e.g., Figure S8).

Exceedances of ISQGs for PAHs have also become more frequent over time in lakes sampled for regional monitoring initiatives (Figure S9), but, to our knowledge, these analyses have not yet appeared in another peer‐reviewed publication. Some of these increases may be attributed to OSIA, but forest fires may also play a role. Retene, although it does not have a guideline, also shows some increases that may be associated with the total area burned in Alberta per year (Figure S10). The increases in sediment retene do not, however, fully explain all increases over time. Future analyses may benefit from augmenting existing approaches to numerically account for forest fires. Analyses that include other covariates may be needed to overcome the challenges of defining reference and exposure zones in the OSR where many stressors are nonpoint and/or diffuse, but similar to other potential studies, the decision criteria needed to initiate this work have not been articulated.

Guidelines have also been used to evaluate metal concentrations in lakes. In sediment cores collected from 21 lakes, guideline exceedances of metal concentrations in sediment were observed in three: RAMP 464 (also called L60), Pushup (also called 2014‐C), and SE22 (Cooke et al., 2017; Figure S1). These exceedances showed neither temporal nor spatial associations with oil sands development and no exceedances were observed at NE13 and NE20 (Cooke et al., 2017). A lack of spatial association with OSIA was also repeated in Hg measured in lake water compared to Alberta Surface Water Quality guidelines (Emmerton et al., 2018). In contrast, the concentrations of total Hg in all samples of lake sediment cores were above the ISQG in NW Saskatchewan (Laird et al., 2013). In sum, these data suggest that some biological impacts may occur in lakes of the minable region, but they would not be common.

#### Biomonitoring in lakes

##### Tissue concentrations

Relative to the physicochemical status, there has been little reporting of tissue concentrations of CoCs from organisms residing in lakes. While not all compounds bioaccumulate to the same degree, the concentrations of parent PAHs and alkylated PACs in tissues of organisms captured in lakes have only been reported in the peer‐reviewed literature in a single recent study (Brunet et al., 2020). This study was performed in Cold Lake and found that benzo*(a)*pyrene (BaP) concentrations were not detectable in lake trout (*Salvelinus namaycush*) or lake whitefish (*Coregonus clupeaformis*) tissues and were below the 2.0 ng/g wet weight consumption limit used in Evans et al. (2019). However, Brunet et al. (2020) found greater concentrations of total PAHs in fish captured in Cold Lake compared to fish caught in the Athabasca and Slave Rivers and, similar to “comparable locations,” increased the lifetime cancer risk from 1 in 10 000 to 1 in 100 000 in humans consuming these tissues (Brunet et al., 2020). Halogenated organics have also been detected in organisms collected from Saline Lake, Burnt Lakes, and Lac La Biche (Xia et al., 2019). A potential causal relationship with oil sands development was not examined, and there are no consumption guidelines for these halogenated compounds.

Elemental concentrations have also been measured in the tissues of fishes residing in lakes. In Cold Lake, the tissue concentrations of Pb, Hg, and As were below Federal consumption guidelines (Brunet et al., 2020). No exceedances of consumption guidelines of Hg were found in additional collections of aquatic species harvested by community members 165 km southwest of Fort McMurray and adjacent to an enhanced (nonthermal in situ) recovery facility (Canadian Natural Brintnell; Golzadeh et al., 2020). The concentration of Hg in muscle tissue of lake trout captured in Namur Lake was higher in 2007 compared to 2000, but similar increases have also been found in lakes in the North West Territories (Evans & Talbot, 2012). There were no consumption limits of walleye for adult men in Lake Athabasca as of 2019 (based on data from 2014), but there are limits for women and children (Government of Alberta, 2019).

##### Status of organisms residing in lakes

While less common than in streams, the status of organisms residing in lakes has been assessed. Because many lakes in the region are fishless, benthic macroinvertebrate surveys are favored, but information from these studies is rare (e.g., Parsons et al., 2010a, 2010b). These analyses reveal no indication of toxic responses related to the development of OS irrespective of the focal contaminants or indicators (Anas et al., 2014, 2017; Neville et al., 2014; Parsons et al., 2010a, 2010b); however, only a single study available as gray literature examined lakes within the “high‐deposition zone” adjacent to the main Suncor and Syncrude stacks. In that study, the abundance and richness of benthic invertebrates in Shipyard Lake increased over time, but equitability (Simpson's Evenness Index) decreased (RAMP, 2016). Results from most other sites (except Christina Lake), such as Kearl (also known as RAMP 418), McClelland, Gregoire, and Isadore's Lakes, also suggested good ecological status (RAMP, 2016; Figure S1).

In contrast to collections of living organisms in streams, studies from the OSR have used lake coring to reconstruct planktonic communities and examine changes over time and space. This technique, recommended in the early reviews (e.g., Donahue, 2011; Dowdeswell et al., 2010) to demonstrate ecological status, suggests that primary productivity has increased over time in most studies of regional lakes (e.g., Hazewinkel et al., 2008). The increases in primary productivity appeared irrespective of proximity to oil sands development (Laird et al., 2013; Mushet et al., 2017) and were also found in lakes with the greatest exposure to atmospherically deposited materials, lakes NE13 and NE20 (Kurek et al., 2013; Summers et al., 2016). Researchers suggest that the increases in productivity are associated with regional climate change (Kurek et al., 2013; Laird et al., 2013, 2020; Libera et al., 2020; Mushet et al., 2017; Summers et al., 2016, 2017). While those general patterns are apparent, some lakes show declines in inferred primary productivity in the most recent core sections and one (P13) shows a decline since ~2000 (Figure S11).

While some of this research did not find a relationship between nutrients deposited in the winter (Summers et al., 2016), other research has observed changes in bogs (Wieder et al., 2019). Consequently, the regional deposition of nutrients may play a role (Scott et al., 2010), but the relationship may be challenging to demonstrate in some study environments.

#### Recommendations for lake studies

Much has been reported on the status of regional lakes surrounding oil sands facilities in the last decade, but specific recommendations can be made to improve research in the OSR. First, some testable hypotheses can be evaluated either using existing data or with (future) reformatted and refocused field collections. These hypotheses include a role or petcoke dust compared to its combustion and can be evaluated by sampling near the Horizon Mine (which has a growing petcoke pile but does not combust this material on‐site) and collections before and after Suncor's process change. The role of other fuels or industrial practices can also be tested; Kearl was opened in 2013 and has no bitumen upgrading equipment on site. As demonstrated in snow, the utility of information from routinely reported industry production data can be further explored to better understand the diverse exposure environments.

Additional areas potentially requiring attention are also apparent. The toxicological status of organisms living in local lakes, the chemical composition of their tissues, understanding the diversity of chemical profiles within and among source materials, including natural and anthropogenic covariates, further examination of Namur Lake, and reconciling differences in PAH ratios in surficial sediments and cores may require dedicated effort. The development of standard and relevant criteria and tools or deliberate use of spatial statistics may also be beneficial.

### Status of streams

#### Water levels and flow

Alterations to surface waters, including stream realignments, road construction, wetland removal, hydrological isolation of project areas, and culvert construction, are known stressors associated with OSIA (Fennell & Arciszewski, 2019; Volik et al., 2020). Recent work suggests that linkages between climate and hydrology are diminished in watersheds with a greater degree of industrial development (Alexander et al., 2020), which may affect the dispersal of contaminants (Cooke et al., 2017), but few studies have addressed the effects of OSIA on streamflow. Among the major tributaries of the Athabasca OSR, discharge parameters have been compared in the Muskeg, Steepbank, Firebag, and Christina Rivers, but any linkages with OSIA were not clear (Schwalb et al., 2015). Similarly, while landscape alteration may also influence flows in the Athabasca River and industry does withdraw water (Andrishak & Hicks, 2011; Pavelsky & Smith, 2008, 2009; Schindler & Donahue, 2006), little data support a discernible, or substantive role of OSIA on water levels downstream of development (Bawden et al., 2014; Dubé & Wilson, 2013; Squires & Dubé, 2013; Squires et al., 2010). Instead, the changes observed over time may be driven by climatic variability (Monk et al., 2012; Sauchyn et al., 2015).

#### Physicochemical status of streams

##### PACs, elements, and nutrients

Studies in the mainstem Athabasca and its tributaries commonly measure the concentrations of elements and organic compounds. Researchers have shown differences in the concentration of chemical endpoints between upstream (reference) and downstream (exposure) sites in the mainstem Athabasca and its tributaries across CoCs (Donner et al., 2017; Droppo et al., 2018; Evans et al., 2019; Glozier et al., 2018; Kelly et al., 2009, 2010; Shotyk et al., 2017; Timoney & Lee, 2009; Wang et al., 2014). While researchers also suggest that the concentrations at exposed locations are lower than those in urban and industrialized areas and are comparable to other freshwater environments (Evans et al., 2019), some studies also commonly show no differences in CoCs. For example, 43% of metals measured under the JOSM between 2012 and 2015 showed no differences in concentration from upstream to downstream in the mainstem Athabasca (Glozier et al., 2018). Specific disagreements are also common. In contrast to spatial increases from upstream to downstream in the concentration of dissolved Se in the Athabasca River observed by Glozier et al. (2018), Donner, Cuss, et al. (2018), found no differences in this endpoint.

While differences in environmental indicators are often reported between locations, the role of industrial activity is not always clear (Akre et al., 2004; Alexander & Chambers, 2016; Arciszewski et al., 2018; Birks et al., 2017; Droppo et al., 2018; Evans et al., 2019; Gerner et al., 2017; Guéguen et al., 2011; Schwalb et al., 2015). These challenges in interpretation may be driven by several factors, including how data are analyzed and sample sizes, but the most important challenge affecting stream studies in the OSR is the potential confounding effects of natural stressors, especially in studies using spatial comparisons of downstream exposure sites to upstream references. In the OSR, the stream sites most likely to be designated as exposed, including those influenced by atmospheric deposition (Arens et al., 2017; Seitz et al., 2013), are also often influenced by bitumen, or other naturally occurring stressors, such as saline groundwater or hydrocarbons from other oil‐bearing formations in the region (Alexander et al., 2020; Arciszewski et al., 2018; Bawden et al., 2014; Bicalho et al., 2017; Birks et al., 2017; Conly et al., 2007; Donner, Bicalho, et al., 2018; Droppo et al., 2018, 2019; Dubé & Wilson, 2013; Evans et al., 2019; Gerner et al., 2017; Gibson et al., 2013; Glozier et al., 2018; Gue et al., 2018; Guéguen et al., 2011; Headley et al., 2005; Hebert, 2019; Hein & Cotterill, 2006; Kilgour, Mahaffey, et al., 2019; Sauchyn et al., 2015; Shakibaeinia et al., 2017; Squires et al., 2010; Yi et al., 2015); this phenomenon has been a significant challenge during freshet and is specifically discussed below (see *Pulses of spring melt* section). In some rivers, such as the Athabasca, there can also be challenges with the confounding influence of cross‐channel effects and the Fort McMurray wastewater treatment plant (Culp et al., 2018, 2020). Overlapping and, in some cases, interacting natural and anthropogenic signals, including changes in flooding frequency, local land disturbances, declines from global sources, and atmospheric deposition may also be apparent in at least one floodplain lake within 1 km of Suncor's Basemine (Klemt et al., 2020).

While many of the studies examining the physicochemical status of streams are unable to definitively separate natural and anthropogenic influences, some patterns suggesting the influence of the former have been identified. Similar to, but more temporally resolved than observations from lakes (e.g., Cooke et al., 2017), there is evidence suggesting that early phases of mine development, such as site preparation and construction, influence the water quality of streams (Alexander & Chambers, 2016; Schwalb et al., 2015). Additionally, using data from consistent sampling locations, temporal studies in the Athabasca River and its floodplain lakes suggest that deposition of V from global sources may be occurring but this conclusion is uncertain and the patterns could instead be related to natural processes (Arciszewski et al., 2018; Klemt et al., 2020). These data, along with study designs favored in lakes and bogs, suggest that adopting these designs in streams may more easily reveal industrial influence compared to designs using upstream–downstream comparisons.

While some site‐specific changes have been identified, not all known events were reflected in analyses. Signals associated with known events, such as the discharge of waters from the “Shell test pit” in the late 1970s (1976–1978; Akena, 1979) and site preparation of the Jackpine Mine in 2006 and Kearl Mine in 2008, were not identified in previous work (Alexander & Chambers, 2016). The lack of differences during the construction of the Jackpine and Kearl mines may be related to any improvements in mitigation practices with greater experience or with more stringent regulations. Additionally, associations between the degree of development in a watershed (Figure S12) or proximity to Suncor and Syncrude's upgrading complexes in patterns of PAC concentrations in rivers (Evans et al., 2016) have not been demonstrated, but few studies have statistically examined these types of relationships.

Nutrients have also been evaluated in the Athabasca River. Measurements of P and N suggest that both are increasing in the Athabasca River over time in the OSR (Dubé & Wilson, 2013; Glozier et al., 2018; Squires et al., 2010). Analysis of data from shorter periods suggests that total phosphorus may have stabilized (2000–2014; Glozier et al., 2018) and, consistent with earlier conclusions (Scrimgeour & Chambers, 2000), suggests a role of the effluent from the Fort McMurray wastewater treatment plant. While there are smaller discharges associated with Oil Sands facilities (e.g., RAMP, 2016), workcamps, and Fort MacKay, their contributions have not been thoroughly examined in the peer‐reviewed literature.

##### Pulses of spring melt

Pulses of contaminant‐laden spring melt is another hypothesized effect of OSIA on the ambient aquatic environment (Alexander et al., 2017). However, research has not explicitly traced CoCs emitted from oil sands facilities, deposited in snow, and transported to streams such as Hg (Wasiuta et al., 2019). Rather than migrating to flowing streams, some of the CoCs may be retained in lakes (e.g., Cooke et al., 2017); bogs (e.g., Shotyk et al., 2016); uplands (Landis, Studabaker, et al., 2019); areas of slow water flow, such as beaver ponds, pools, and muskeg, or are incorporated into groundwaters. As with other studies in streams, however, an influence of spring melt laden with contaminants deriving from industrial activities may be masked by in‐stream processes or natural inputs of metal and organic‐rich groundwaters (Birks et al., 2017; Evans et al., 2019; Gerner et al., 2017; Yi et al., 2015). However, in some areas, different aspects of industrial development may also interact and reduce signals of atmospheric deposition in streams (Alexander et al., 2020). Restrictions of study designs to smaller spatial scales and the development of dedicated integrated designs may resolve this challenge.

**Figure 5:**
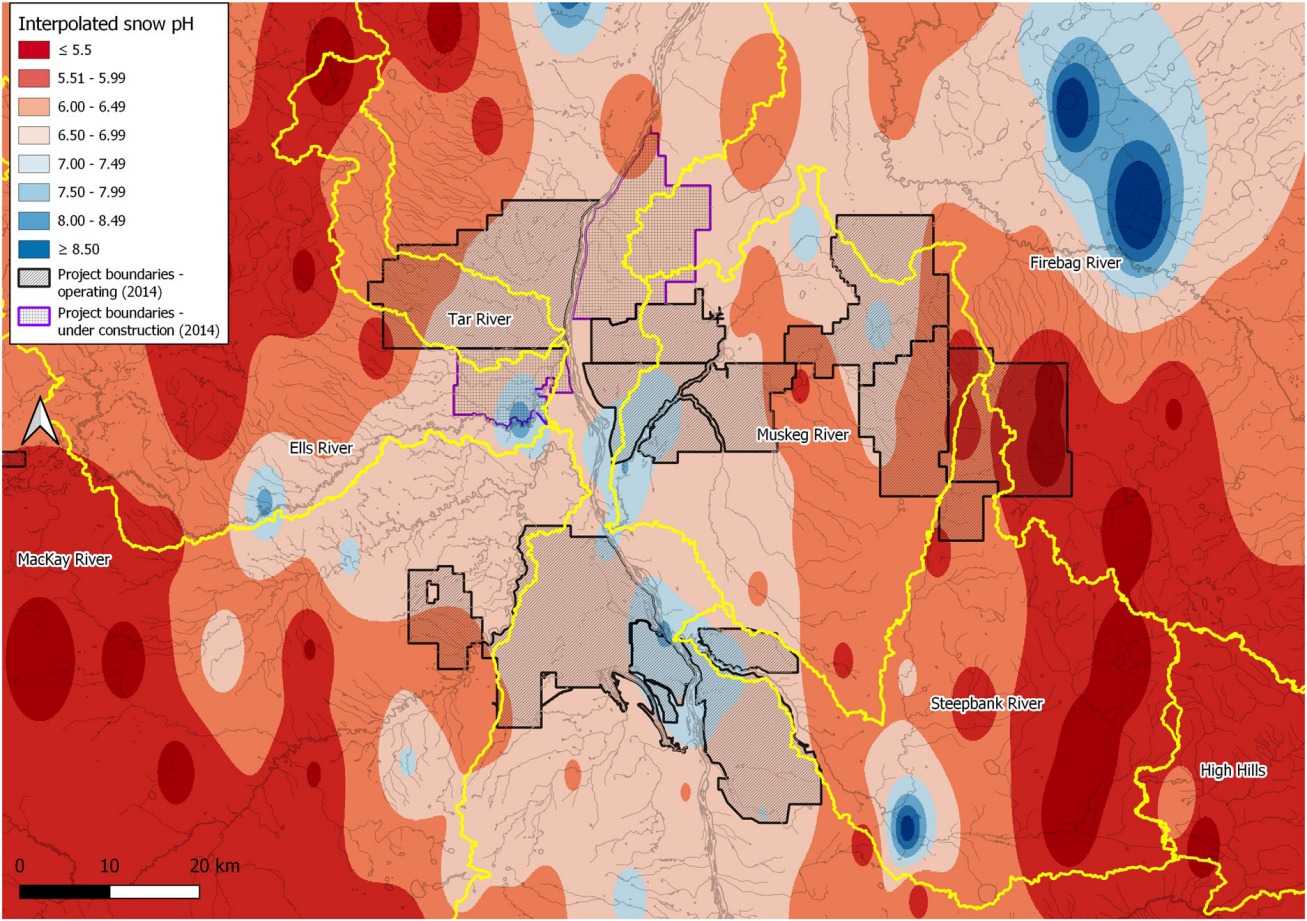
Interpolated surface (inverse distance weighting; QGIS 3.16) of pH in snow samples collected in 2014; distance coefficient = 2; data available from OSM Portal: https://www.canada.ca/en/environment-climate-change/services/oil-sands-monitoring/monitoring-water-quality-alberta-oil-sands.html; also shown are the major watersheds in the minable region obtained from http://www.ramp-alberta.org/RAMP.aspx. High Hills watershed was delineated using Upslope Area in QGIS (3.16)

Acidification of streams via a pulse of low‐pH snow driven by industrial activity has also been hypothesized (Schindler, 2013) and is the focus of some work in the OSR. In analyses of water quality measured between 1989 and 2014, waters with a pH less than 6.5 were observed in 4% of observed snowmelt events and may be associated with OSIA (Alexander et al., 2017). However, other data collected in regional monitoring are not spatially consistent with this suggestion. In 2014, the pH of snow was higher in areas closer to mines, suggesting that OSIA alkalizes, not acidifies, snowmelt (Figure 5). Greater base cations are also observed closer to mines in other studies (Fenn et al., 2015). The detection of more frequent acidification events in the original work may be associated with the addition of sampling at some reference sites after 2010, such as the High Hills River, or how “impact” and “reference” labels are appended to entire rivers (Alexander et al., 2017; Figure 5). Formal criteria may be required to address site naming, especially if studies expand beyond medium‐centric themes, like air, water, and land.

The potential effects of spring melt pulses have also been evaluated using laboratory bioassays. Recent work published by Parrott et al. (2018) suggests that melted snow collected from sites within a 7 km radius of mine footprints was associated with reduced survival of fathead minnow (*Pimephales promelas*) exposed in the laboratory. However, when water was also collected from tributaries during snowmelt and used in exposures, no significant effects in fish embryos were observed in the laboratory testing (Parrott et al., 2018). Snow collected more than 25 km from surface mines also did not significantly affect larval fish (Parrott et al., 2018). These data confirm risks to exposed organisms, but as discussed by the authors, there were potential challenges of when tributary spring melt samples could be safely collected and what degree of exposure those samples captured; in one experiment, 50%–80% of contaminants were released in the first 30% of snowmelt (Johannessen & Henriksen, 1978). The effects of a pulse of acidic waters into slow‐flowing areas with organic soils, such as the headwaters of the Muskeg River, are also not specifically known.

##### Oil sands process‐affected water (OSPW)

The potential occurrence of OSPW outside of containment in surface waters is a primary concern in the region (Hazewinkel & Westcott, 2015; McQueen et al., 2017; Schindler, 2010; Timoney & Lee, 2009). Compliance reporting suggests that infiltration of OSPW into groundwaters adjacent to tailings ponds is common (Fennell & Arciszewski, 2019), and researchers also suggest that OSPW may be present in the interstitial (and upward‐flowing) waters in sediments beneath the Athabasca River adjacent to Suncor's (former) Pond 1 (Frank et al., 2014a, 2014b; Hewitt et al., 2020). While that research did not include samples of Athabasca River water and suggests that any OSPW reaching it would be diluted, other researchers contest the results (Yi et al., 2014). Other work is also divided. Some suggest that their data show no definitive observations of OSPW outside of containment in the Athabasca River water (Ahad et al., 2013; Ross et al., 2012; Sun et al., 2017), while others contend the opposite (i.e., Kelly et al., 2010; Timoney & Lee, 2009). Part of this disagreement is likely associated with the absence of published data showing OSPW in samples of water collected from the Athabasca River proper (e.g., Sun et al., 2017), but that upward‐flowing interstitial waters in the sediments beneath the Athabasca may contain OSPW (e.g., Frank et al., 2014a) and if the latter constitutes proof that this substance is present in the River.

Studies have also examined the potential seepage of OSPW into the Athabasca River tributaries. Researchers suggest that OSPW may be present in two tributaries, which have both been realigned during mine construction and are within 500 m of tailings ponds and McLean Creek and Lower Beaver River (Fennell & Arciszewski, 2019; MacKinnon et al., 2005; Ross et al., 2012; Sun et al., 2017). In contrast to evidence of OSPW in monitoring wells between the Mildred Lake Settling Basin and the Lower Beaver River (also called Lower Beaver Creek), compliance reporting suggests no evidence of OSPW between McLean Creek and Suncor's ponds at the Millennium Mine (Fennell & Arciszewski, 2019). In other tributaries (Muskeg, MacKay, and Steepbank Rivers) where the consensus view is that OSPW is not present and patterns are driven by natural groundwaters (Ross et al., 2012; Roy et al., 2016; Sun et al., 2017), other researchers suggest that their data may show evidence of seepage (Arens et al., 2017).

Disagreements regarding the occurrence of seepage in both the Athabasca River and its tributaries are additional examples of the challenges of identifying the influence of anthropogenic activities in the presence of natural confounding factors. Groundwaters are expected to be the conduit connecting tailings ponds and surface water bodies (Fennell & Arciszewski, 2019), but groundwaters naturally in contact with bitumen are commonly observed in the region, including undeveloped areas more than 10 km from tailings ponds (Sun et al., 2017). Additionally, groundwaters with natural exposure to bitumen can also occur close to the borders of tailings ponds where groundwaters influenced by OSPW may also be present (Roy et al., 2016). While the interference of natural patterns often obscures any conclusive results of anthropogenic influence (Broughton, 2020; Gibson et al., 2013) it also partially fuels the continued controversy of the potential seepage of OSPW into surface waters (Fennell & Arciszewski, 2019). Much of the controversy regarding OSPW seepage in surface waters may, however, be settled with families of indicator compounds enriched in OSPW compared to natural groundwaters (Hewitt et al., 2020). However, this approach depends on the enrichment of indicator compounds via water reuse and recycling in the oil sands process and was originally demonstrated using data from the original two tailings ponds (Hewitt et al., 2020); the utility of this technique for newer ponds holding water with a lower degree of recycling has not been evaluated.

#### Exceedances of environmental quality guidelines in streams

Contaminants of concern measured in either water or sediments of streams are commonly compared to environmental quality guidelines. Droppo et al. (2018) reported exceedances in benzo*(a)*anthracene, benzo*(a)*pyrene, chrysene, fluorene, naphthalene, and phenanthrene in suspended sediments collected from the Ells and Steepbank Rivers in 2013 and 2014 (Table 2). However, similar to the concentrations of some metals, such as Cd in lake sediments (Cooke et al., 2017; Figure S5), there are quality issues with these data; the detection limits for acenaphthene, acenaphthylene, and dibenz*(a,h)*anthracene were greater than the ISQG (Table 2) and many PAHs were reported at the detection limit of 20 ng/g. The exceedance rates reported here do not consider the potential for temporal and/or spatial autocorrelation, but the similarities by which compounds exceed sediment guidelines suggest a greater role of natural sources rather than OSIA, which differs greatly between these two tributaries (Figure S12). However, similar to other studies, exceedances of ISQGs in the Ells in 2013 and 2014 could be associated with site preparation for the Joslyn North Mine.

**Table 2:** Number of guideline exceedances among total sample sizes (*x*/*n*) for 12 parent PAHs measured in the Steepbank (STB) and Ells Rivers in 2013–2014 using Phillips Tubes; data are available from https://www.canada.ca/en/environment-climate-change/services/oil-sands-monitoring/monitoring-water-quality-alberta-oil-sands.html, published in Droppo et al. (2018)

PAH	ISQG (ng/g)	PEL (ng/g)	DL (ng/g)	ISQG		PEL	
				STB	Ells	STB	Ells
Acenaphthene^a^	6.71	88.9	20	13/13	11/11	0/13	0/11
Acenaphthylene^a^	5.87	128.0	20	13/13	11/11	0/13	0/11
Anthracene	46.90	245.0	20	0/13	0/11	0/13	0/11
Benzo*(a)*anthracene	31.70	385.0	20	1/13	0/11	0/13	0/11
Benzo*(a)*pyrene	31.90	782.0	20	4/13	4/11	0/13	0/11
Chrysene	57.10	862.0	20	4/13	7/11	0/13	0/11
Dibenz*(a,h)*anthracene^a^	6.22	135.0	20	13/13	11/11	1/13	3/11
Fluoranthene	111.00	2355.0	20	0/13	0/11	0/13	0/11
Fluorene	21.20	144.0	20	5/13	6/11	1/13	3/11
Naphthalene	34.60	391.0	20	3/13	4/11	1/13	2/11
Phenanthrene	41.90	515.0	20	2/13	4/11	1/13	1/11
Pyrene	53.00	875.0	20	0/13	0/11	0/13	0/11

Abbreviations: ISQG, Interim sediment quality guideline; PAH, polyaromatic hydrocarbon; PEL, probable effects level.

^a^ Compounds with detection limit exceeding the Guideline.

Comparisons of sediment PAHs to guidelines were also reported in Evans et al. (2016). Among the sediments collected from tributaries over multiple years, exceedances were observed at most locations, except MUR‐D3 and JAC‐D2, where none were observed (Table 3). Among these sites, JAC‐D2 is closest to the high‐deposition zone defined in other studies (Manzano et al., 2016). The most exceedances are observed in the Ells, Muskeg, and Tar Rivers (Table 3). Similar to data from lakes, the rate of exceedances does not follow the proportion of landscape disturbance in these watersheds when all are considered (Figure S12). However, there is some evidence to suggest that exceedances of individual PAHs are more common over time in the Tar River, which could be related to the degree of development in that basin, but also reinforces potential challenges with deriving regionally applicable conclusions in the OSR from the study designs used (e.g., Droppo et al., 2018). Among the exceedances reported in Droppo et al. (2018), many of the compounds also exceeded guidelines in Evans et al. (2016).

**Table 3:** Percentage of guideline exceedances (interim sediment quality guidelines) of polyaromatic hydrocarbon (PAH) exceedances (%) for sites with long‐term records from the minable region; data published in Evans et al. (2016), available from http://www.ramp-alberta.org/RAMP.aspx

PAH	1998–2015	2002–2015	2000–2015	1997–2015	2006–2015	2000–2015	2003–2015	2008–2015	1997–2015	1998–2015
	ELR‐D1	FIR‐D1	FOC‐D1	JAC‐D1	JAC‐D2	MUR‐D2	MUR‐D3	BER‐D2	POC‐D1	TAR‐D1
Acenaphthene	15.38	0.00	0.00	0.00	0.00	7.14	0.00	0.00	0.00	15.38
Acenaphthylene	0.00	0.00	0.00	0.00	0.00	0.00	0.00	0.00	0.00	0.00
Anthracene	0.00	0.00	0.00	0.00	0.00	0.00	0.00	0.00	0.00	0.00
Benz*(a)*anthracene	15.38	0.00	0.00	0.00	0.00	7.14	0.00	0.00	0.00	15.38
Benzo*(a)*pyrene	7.69	0.00	0.00	0.00	0.00	0.00	0.00	0.00	0.00	15.38
Chrysene	100.00	12.50	72.73	0.00	0.00	57.14	0.00	0.00	30.00	30.77
Dibenz*(a,h)*anthracene	46.15	12.50	54.55	0.00	0.00	28.57	0.00	0.00	18.18	30.77
Fluoranthene	0.00	0.00	0.00	0.00	0.00	0.00	0.00	0.00	0.00	0.00
Fluorene	0.00	0.00	0.00	0.00	0.00	0.00	0.00	0.00	0.00	0.00
Naphthalene	0.00	0.00	0.00	0.00	0.00	0.00	0.00	0.00	0.00	0.00
Phenanthrene	15.38	0.00	0.00	0.00	0.00	7.14	0.00	0.00	9.09	23.08
Pyrene	38.46	0.00	9.09	0.00	0.00	14.29	0.00	0.00	0.00	0.00

*Note*: Site locations are shown in Figure S1.

The concentrations of elements in water and sediments are more commonly measured and reported against environmental quality guidelines compared to organics. In the lower Athabasca River, guideline exceedances have been observed in the concentrations of several total metals in water and sediment samples from both the mainstem Athabasca and its tributaries (Kelly et al., 2010; Timoney & Lee, 2009). Exceedances of quality guidelines for elemental concentrations were also commonly observed in 2012–2015 in water samples from the Athabasca River, but many were associated with high sediment loads driven by greater flows (Glozier et al., 2018; Hebert, 2019) and may have been associated with stable minerals (Javed & Shotyk, 2018). In contrast to exceedances observed in the total concentrations of Cd, Ag, and Pb by Kelly et al. (2010) in water samples, concentrations of dissolved metals were as high as 3 orders of magnitude below the United States Environmental Protection Agency's Guidelines for the protection of aquatic life (Shotyk et al., 2017). Historically, exceedances of water quality guidelines in tributaries were observed in 12 of 17 variables examined before development and 9 of 17 afterward, but exceedances of Al, Fe, and Ag became more frequent after the onset of development (Alexander & Chambers, 2016). However, there were periodic discharges of waters from a test pit in the Muskeg drainage between 1976 and 1978 (Akena, 1979) and this may influence the results from the predevelopment period.

Additional challenges with water and sediment quality guidelines, including potential exceedances of natural concentrations of total metals (Headley et al., 2005), prompted Glozier et al. (2018) to recommend the development of site‐specific benchmarks for understanding changes in environmental quality. Site‐specific benchmarks for water quality measurements, such as those accounting for natural environmental covariation and censored chemical data, have been developed for two locations in the Athabasca River (Arciszewski et al., 2018). However, these guidelines are statistical thresholds and not inherently relevant for assessing toxicity (Arciszewski et al., 2018). There is also debate over the relevance of guidelines for the concentrations of total and dissolved metals in water (Shotyk et al., 2016), and studies have identified influences at concentrations up to 100× lower than some guidelines (Gerner et al., 2017).

#### Biomonitoring in streams

##### Tissue concentrations and physiological responses

Tissue concentrations of many CoCs have been measured in organisms residing in streams. Some such as naphthenic acid fraction compounds (NAFCs) have been identified in fish tissues and bile (e.g., Arens et al., 2017), but they are not considered highly bioaccumulative (Van den Heuvel et al., 2014; Redman et al., 2018; Scott et al., 2020; Smits et al., 2012; Young et al., 2011; Zhang, Pereira, et al., 2015), and not all studies show detectable NAFCs in the tissues of fishes (Young et al., 2008, 2011). Any relationship between NAFCs derived from OSIA and the occurrence of these compounds in tissues or bile of resident organisms has not been demonstrated.

Tissue concentrations of PACs are commonly measured in fish. Goldeye (*Hiodon alosoides*) lake whitefish, northern pike (*Esox lucius*), walleye (*Sander vitreus*), and burbot (*Lota lota*) collected in the Athabasca and Slave Rivers contained residues of PAHs in their muscle tissues in patterns consistent with exposure to hydrocarbons (Ohiozebau et al., 2017). This finding is also consistent with other research (Arens et al., 2017; Cash et al., 2000; Evans et al., 2019; Ohiozebau et al., 2016). The role of industrial or other influences, such as wildfires, specifically including the large Richardson Fire in the Spring of 2011, in some of these patterns (e.g., Ohiozebau et al., 2016) is, however, not certain. Regardless of their origin, tissue concentrations of PACs in fish are below applicable guidelines (Evans et al., 2019), and Ohiozebau et al. (2017) further suggested that the additional average lifetime risk to residents in the OSR consuming locally caught fish was less than 1 in 1 000 000 and fell within an acceptable range.

Tissue concentrations of metals have also been measured in fish and invertebrates. Some researchers show increases downstream of developments (Arens et al., 2017; Pilote et al., 2018), but contrasting evidence has also been reported (Donner, Cuss, et al., 2018; Pilote et al., 2018; Shotyk et al., 2019; Sinnatamby et al., 2019). Additionally, while differences consistent with industrial exposures have been found, these studies did not explicitly account for natural changes in bedrock or surficial geology between reference and exposure locations that influence water quality in the OSR (e.g., Akena, 1979; Headley et al., 2001, 2005; Spiers et al., 1989).

Fish tissue Hg is a high‐priority issue in the OSR, but there are disagreements in the published literature. In white sucker captured during spawning (May 2012), Arens et al. (2017) found lower concentrations of Hg in liver tissue of fish from the Muskeg River compared to fish from Calling Lake (0.038 and 0.180 mg/kg, respectively). Mercury concentrations in pooled muscle samples from two species captured in the Athabasca River, spottail shiner (*Notropis hudsonius*) and emerald shiner (*N. atherinoides*), were the highest near oil sands mines (Dolgova et al., 2018). However, the largest upstream to downstream increase in tissue Hg concentration in fishes captured in 2013 (~6‐fold) occurs between the site at the Town of Athabasca and another at the southern margin of the mining footprint. While the elevated tissue concentration of Hg identified at the southern margin of the mining footprint persists until the Tar River (Dolgova et al., 2018), similar to earlier work (Kelly et al., 2010), the specific origin of this difference cannot be identified; a partial, combined, or complete role of municipal effluents or other releases or emissions from the City of Fort McMurray (Lynam et al., 2015) cannot be ruled out.

Other data also suggest little evidence of a local industrial source in Hg of fish muscle (Tendler et al., 2020), but does show that tissues of sport fishes, such as walleye and pike, can exceed consumption and subsistence guidelines (Tendler et al., 2020). Temporally, Hg has been measured in fishes captured in the OSR since 1976. When the size of fish is included as a covariate, concentrations of Hg in the muscle of walleye and lake whitefish decreased between 1984 and 2011 in the reaches of the Athabasca River in the minable oil sands area (Evans & Talbot, 2012). Analyses of the mean Hg in walleye by (Timoney & Lee, 2009) suggest an increase between 1976 and 2006, but this analysis did not account for the confounding influences of fish size, which is a known driver of bioaccumulating substances like Hg (e.g., Kidd et al., 2012). Based on data from 2014, in 2019, there were no consumption limits of walleye for adult men in the Athabasca River, but there are limits for women and children (Government of Alberta, 2019).

Physiological responses of organisms to hydrocarbon and metal exposure have also been measured. Measurements of liver detoxification enzymes (McMaster et al., 2006a, 2020; McMaster, Parrott, et al., 2018; McMaster, Tetreault, et al., 2018; Tetreault et al., 2003, 2020) also suggested exposure and physiological responses to hydrocarbons, but the researchers were unable to link them with an industrial influence. Potent inducers of liver detoxification enzymes were found in the oil sands area and were the highest in the Peace, Athabasca, and Slave Rivers (Cash et al., 2000; Glozier et al., 2018; Parrott et al., 1996), but the profiles of PAHs collected using semipermeable membrane devices suggested that these compounds derived from natural sources (Glozier et al., 2018).

Other physiological measurements have been performed in the OSR. Similar observations have also been made in in vitro steroid production (Tetreault et al., 2020), proteomics (Simmons & Sherry, 2016), and metallothionein (Pilote et al., 2018). Similar to other studies, the presence of confounding influences, such as the geological differences among study locations (e.g., Akena, 1979; Headley et al., 2001, 2005; Spiers et al., 1989), typically obscures any industrial contributions.

##### Physical abnormalities in fish

Potential impacts of oil sands development on the health of organisms, including fishes, and subsequent consumption have motivated much of the research attention in the region (Miall, 2013). While blinded panels were unable to differentiate the taste of steamed samples of fishes captured from three locations in Alberta, including the Athabasca River (Barona et al., 2011), oily and soft flesh of fish has been reported by residents downstream of the oil sands area (Timoney & Lee, 2009) and from Cold Lake (Brunet et al., 2020). Physical abnormalities in fishes have also been noted by residents (Brunet et al., 2020; Miall, 2013; Timoney & Lee, 2009). These data suggest a possible increase in the rates of abnormalities over time, including a roughly fourfold increase in the frequency of fin erosion in migratory fishes in the late 1990s compared to 1987–1991 (Schwalb et al., 2015) and compared to earlier data from Mill et al. (1996) and remained elevated for the duration of the data set (2007–2012; Schwalb et al., 2015). The causes of these observations are not clear, but they highlight some of the challenges of some monitoring in the OSR (Beausoleil et al., forthcoming).

Other work has examined fish abnormalities. Timoney and Lee (2009) report the incidence of internal and external abnormalities in fishes using data from the Regional Aquatics Monitoring Program. The most commonly reported abnormality was the occurrence of parasites (Timoney & Lee, 2009), and later work has suggested that the parasite burden in fish and its community composition may be related to land use (Blanar et al., 2016) but also local habitat (Raine et al., 2017).

##### Status of organisms residing in streams

Multiple studies of organisms residing in streams have been performed in the OSR. Among them, spatial designs are popular and some have found differences between sites consistent with the potential influence of industrial activities but also with natural or other confounding factors (Arens et al., 2017; Culp et al., 2018; Farwell et al., 2009; Gerner et al., 2017; Pilote et al., 2018; Tetreault et al., 2003). Some studies also suggest a potential role of sewage inputs (Culp et al., 2020; McMaster, Tetreault, et al., 2018; Simmons & Sherry, 2016), although the influence of this stressor has not always been highlighted (Lavoie et al., 2011).

Spatial analyses have also been performed over multiple years, including during construction and ~10 years after the opening and expansion of the Millennium mine (Tetreault et al., 2020). This study found consistent patterns of change, suggesting that the encroachment of industrial activity did not affect fish (Tetreault et al., 2020). While a lack of spatial differences has also been observed in some endpoints from fish collected in the mainstem Athabasca (McMaster et al., 2006b; McMaster, Tetreault, et al., 2018), the lack of changes in the Steepbank River over time may be related to the low proportional increase in disturbed area between the study periods (Figure S12) or the performance of on‐site mitigation.

**Figure 6:**
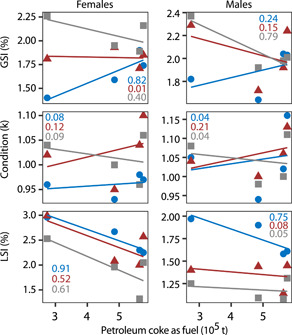
Mean relative gonad weight (gonadosomatic index; GSI; %), body condition (*k*), and relative liver weight (liver‐somatic index; LSI; %) of female and male slimy sculpin (*Cottus cognatus*) captured at the lower Steepbank Location (STR‐L; blue circles), middle Steepbank site (STR‐M; red triangles), and upper Steepbank (STR‐U; gray squares) locations from 2010 to 2013 (data provided in Tetreault et al., 2020) compared to Suncor petroleum coke fuel data (AER, 2021) from January to September of the respective year; variability of means and 95% CIs of regression lines have been omitted for clarity. Fishing locations shown in Figures 2 and S1; values within panes show *R* ^2^ for STR‐L, STR‐M, and STR‐U (top to bottom, respectively)

Despite the lack of consistent differences between the Steepbank River sites over time, additional analysis was performed using these data (Tetreault et al., 2020) and industrial performance data (AER, 2021). No regressions were statistically significant at the 0.3% level (Bonferroni adjustment for 18 comparisons; *p*≥0.081), but they do suggest a relationship between industrial activity on some mean indices of fish health, including GSI, LSI of females and LSI of males captured at the STR‐L from 2010 to 2013 (Figures 6 and S1). However, some of these data are also inconsistent with a relationship expected with proximity to the Suncor Basemine (e.g., GSI of males captured at STR‐U is more strongly related to the index of industrial activity than for males at STR‐L; Figure 6). Other data (LSI of females) also suggest both natural upstream‐to‐downstream changes coupled with influences of industrial activity that may extend to the SR‐U location (Figure 6). There were also no consistent spatial differences in these fish reported in the primary literature (Tetreault et al., 2020), few annual means were available (nor were industry data from before 2009 available at the time of preparing this manuscript), and the industry index is likely colinear with other descriptors of industrial influence, such as landscape disturbance and the mass of petroleum coke stockpiles.

Despite these potential issues, the *R* ^2^ between fish metrics and an index of industrial activity is compelling and, along with the snow deposition analyses described above, suggest that using facility data may enhance understanding, tracking, and predicting environmental responses to the operation of oil sands facilities in the OSR. These data further suggest the greater applicability of site‐specific analyses compared to upstream–downstream designs and also the potential utility of industrial facility data in resolving the long‐standing challenges of the spatial confounds of natural bitumen exposure adjacent to mines. However, more analyses are required to address the potential limitations of the preliminary analysis that we have presented here.

The reanalyses of fish described above suggest the utility of site‐specific analyses over time, and some studies from the OSR have also used this approach. Fish fences were installed in the Muskeg River in 1976, 1977, 1995, 2003, 2006, and 2009 to intercept adult fish migrating into this tributary to spawn (Schwalb et al., 2015). Sharp declines in four of the five most commonly captured species (white sucker, longnose sucker, mountain whitefish, Arctic grayling, but not northern pike) were observed over time, but the largest decreases occurred between 1977 and 1995. The estimated degree of land disturbance in the Muskeg Basin increased from 0.19 in 1977 to 2.81% in 1995 (ABMI, 2018; Figure S12) and comprised exploratory activities (including the Alsands/Shell test pit; Akena, 1979), road construction, and lumber harvesting (ABMI, 2018; Figures S12 and S13). However, there were no commercial oil sands facilities in the Muskeg drainage between 1977 and 1995. An association with the initial changes in spawning fish populations from 1977 to 1995 (or in their persistence until at least 2009) and any OSIA, including emissions from Syncrude's Mildred Lake and Suncor's Basemine, is uncertain. Additionally, some spawning fish migrate from Lake Athabasca, suggesting differing exposure histories both within and among some species (Arciszewski, Munkittrick, Kilgour, et al., 2017 and references therein). Although relationships specifically between oil sands development and changes in spawning fish in the Muskeg basin are not clear, the changes could be associated with low levels of physical disturbance.

Data supporting the rapid onset to low levels of disturbance have been observed in other studies of fish examined over time. The mean condition of six species of fish captured in the Athabasca fish inventory decreased by roughly 6% between the 1970s and 1996–2000 (Schwalb et al., 2015). There are, however, potential challenges with interpretation of these data. No sample sizes or variability were reported for the condition (K) of female walleye captured in the 1970s (Bond & Berry, 1980; Schwalb et al., 2015). Additionally, in the 1970s, fish were collected using bottom‐set gillnets deployed in slow flow areas, while fish collected afterward were captured using boat electrofishers (Bond & Berry, 1980; Schwalb et al., 2015). While these challenges make the differences difficult to interpret, if real, the changes reported in the mean condition of fish in 1996–2000 may support a rapid onset of change to oil sands development also observed in other work (e.g., Alexander & Chambers, 2016).

In contrast, other information from biological indicators also suggests improvements in ecological conditions. While the declines in most species passing through the Muskeg River fish fences persisted until at least 2009 (Schwalb et al., 2015), in that year, the largest recorded spawning run of white sucker was documented. While additional analyses also suggest that the condition of walleye was lower than expected in 1997–1998 (e.g., Schwalb et al., 2015), but the condition was higher than expected in 8 of 14 years after 1998 in a parallel analysis (Arciszewski, Munkittrick, Kilgour, et al., 2017). Additionally, the condition (K) of walleye collected in 2013 was similar to 1977 (Arciszewski, Munkittrick, Kilgour, et al., 2017), also suggesting potential improvements in ecological conditions. These changes may also be related to reduced commercial fishing in the late 2000s (if related to effort and not stock collapse) and changes in white sucker numbers. Some of the recent increases may, however, also be related to sewage inputs from the growing population of Fort McMurray (Arciszewski, Munkittrick, Kilgour, et al., 2017) or with treatment improvements at this facility.

#### Recommendations for studies of streams

Spatial studies performed using upstream reference compared to downstream exposure sites have been a staple in the OSR for decades. Despite this emphasis, few, if any, of these designs have unequivocally demonstrated the influence of OSIA. Among other study environments like lakes, site‐specific analyses are preferred and have demonstrated their utility (Cooke et al., 2017; Kurek et al., 2013; Summers et al., 2016). Area‐specific studies of snow (e.g., Gopalapillai et al., 2019) have also shown the utility of examining fixed sampling areas over time. Studies of streams have been slow to adopt this approach, but among the studies suggesting an influence of OSIA, a site‐specific approach has been used (Alexander & Chambers, 2016; Schwalb et al., 2015). While a site‐specific approach for examining change in studies of streams has not been widely adopted, coupling these collections with some environmental covariates (Kilgour, Munkittrick, et al., 2019) and industrial performance data may allow influence to be tracked and to overcome the persistent challenge of natural confounds in the OSR (Arciszewski, Munkittrick, Scrimgeour, et al., 2017), and the false‐negative problem common in monitoring studies (Munkittrick et al., 2019). Additionally, these approaches can be combined with a priori integrated monitoring designs (e.g., Arciszewski et al., 2021) to address the potential linkages between known stressors and measurement endpoints, such as atmospheric deposition and physicochemical status, rather than a posteriori techniques already used (Wasiuta et al., 2019). The studies could be further refined by focusing on streams in high‐deposition areas, such as the mouth of the Steepbank River. Accounting for other industrial stressors, either generic (e.g., land disturbance) or specific (e.g., petroleum coke stockpiling), may also provide insight into the state of the environment.

Similar to studies of lakes, additional recommendations can also be made. These include better characterizing atmospheric deposition and exposure environments adjacent to multiple facilities. Analysis of data already collected from areas developed during the last 10 years, such as the lower Ells (construction of the Joslyn North Mine was suspended in May 2014) and the beginning of mining at the Kearl Mine (April 2013), may also yield important insights. Other mine expansions are also currently in review or have received regulatory approval. Analyses of data may also benefit from including environmental covariates (Gerner et al., 2017) and, more broadly, integrating more sophisticated statistical approaches across media in partially integrated designs (Arciszewski et al., 2021) to identify and track environmental status in the streams of the OSR.

## CONCLUSION

The scope of regional monitoring in the OSR covers a large area, many environments, multiple indicators, study approaches, experimental designs, statistical analyses, objectives, and various periods of time. Among aquatic habitats, the majority of the research has been carried out in or adjacent to the minable region paralleling historical emphases and regional concerns of open‐pit mining compared to in situ operations. The majority of this work has examined the occurrence of CoCs, their concentrations, and patterns over space and time. Studies typically show higher concentrations and loadings of CoCs adjacent to mines, but the patterns are not spatially uniform, indicating the influence of multiple sources, such as mining dust. Deposition from global sources may also be apparent. Changes in the loading of CoCs to snow in the zone of highest deposition (within ~8 km Suncor's coke stockpiles and upgrading complex) have decreased over time. In some lakes, such as NE20, the atmospheric deposition rate of PACs is increasing while the deposition rate of some metals is decreasing, suggesting different sources. Guideline exceedances in water and sediments from lakes are also apparent, but these are not always associated with oil sands development and there are no indications of toxicity from lake sediment cores irrespective of the composition and likely interaction of known and unknown chemicals. Instead, studies in regional lakes suggest greater primary productivity potentially associated with global climate change including those with the greatest degree of chemical influence from OSIA (e.g., NE13 and NE20). Collections of living invertebrates from another location in the high‐deposition zone, Shipyard Lake, also show no indication of toxicity.

Evaluating the status of streams is a high priority of regional monitoring in the oil sands area. Differences between upstream reference and downstream exposed sites are routinely observed across indicators, such as chemical and biological endpoints, but the spatial designs often cannot separate natural and anthropogenic influences. However, some differences that may be attributed to OSIA have been found. Typically, these differences are found using data records of more than ~5 years and using site‐specific analyses. In these studies of rivers, there was potential evidence of early site preparation activities on the physicochemical status of streams, but little of operating facilities by any exposure pathway, including deposition of materials transported by the atmosphere. The biological data from rivers were also divided. There may be evidence of persistent effects in numbers of spawning fish and rates of fin erosion, but some species have recovered spawning numbers, are larger, and sentinel fish did not appear to respond to the encroachment of new mines. Our additional analyses at a site within the high‐deposition zone (STR‐L) suggest that there may be a discernible association between local activity and the health indices of fish. However, these data are not conclusive and require further scrutiny and dedicated work.

While general conclusions can be drawn, much of the work remains temporally, spatially, or technically isolated, creating gaps in the information available to assess the status of the aquatic environment. These gaps include a lack of temporal and spatial overlap of available data, the absence of potentially relevant material from the peer‐reviewed literature, and the absence of fully or partially integrated designs and integrated analyses. There are also gaps in how to address the influence of the natural processes, accounting for other anthropogenic stressors, how to define reference and exposure locations, and a lack of decision criteria and undefined standards of evidence used to determine if and when impacts are occurring and consequently to identify research, monitoring, and management priorities.

We also provided some recommendations. These include, but are not limited to, more use of available industry data, emphasis on areas likely to have the greatest exposure to diffuse sources, evaluating any environmental data collected near facilities that have changed operational status (or sampling near facilities that will to establish a baseline of current status), and movement away from a posteriori (e.g., interpretation and analysis) toward a priori forms of integration (partially or fully integrated designs; Arciszewski et al., 2021).

## Supporting information

This article includes online‐only Supporting Information.

Supplemental Information A: Additional written material plus supplemental tables and figures.

Supplemental Information B: Table of reviewed papers.

## Data Availability

No new data were included in this article but some data collected through the Oil Sands Monitoring Program are available via https://www.canada.ca/en/environment-climate-change/services/oil-sands-monitoring.html and https://aws.kisters.net/OSM/applications/login.html?publicuser=Guest#waterdata/stationoverview. Other data appearing in this article are cited throughout.
