# Supplementary material for: A critical review of the ecological status of lakes and rivers from Canada's oil sands region
Source: Integr Environ Assess Manag. 2021 Oct 25;18(2):361–87. doi: 10.1002/ieam.4524 (PMC9298303; doi:10.1002/ieam.4524)
Supplement: Supplementary file 1 — Supplemental Information A: Additional written material plus supplemental tables and figures. [file IEAM-18-361-s002.docx]

Supplemental Information for:

A critical review of the ecological status of lakes and rivers from Canada’s Oil Sands Region

Tim J. Arciszewski^1,*^, Roderick R. O. Hazewinkel^2^, Monique G. Dubé^1, 3^

^1^Environmental Stewardship Division, Alberta Environment and Parks, Calgary, AB

^2^Environmental Stewardship Division, Alberta Environment and Parks, Edmonton, AB

^3^ Current Address: Cumulative Effects Environmental, Inc. Calgary, AB

Integrated Environmental Assessment and Management; DOI: 10.1002/ieam.4524

# ORGANIZING LITERATURE

One hundred and twenty-three papers reporting the results of field studies were identified for this review (Supplemental Figure 14; Supplemental table 2; Supplemental Information B). Between 2009 and 2019, the annual appearance of papers used in this review peaked in 2017 at 20 (Supplemental Figure 14). Of the 123 papers identified for this review, we estimate 43% were funded through Oil Sands Monitoring and the Joint Oil Sands monitoring programs (OSM/JOSM). Fifty-three papers report measurements in rivers and 31 report measurements from lakes. Wetlands (11), snow (8), and areas in the Peace Athabasca Delta (9; including 1 paper in the Slave River Delta) have received comparatively less attention in the peer-reviewed literature. Among the 123 papers identified for this work, 119 provided information relevant for the Athabasca Oil Sands (i.e., minable) region, 7 for the Cold Lake Region, and 4 for the Peace Region.

Information on Pressures, Stressors, Pathways, and Receptors can also be extracted from these papers (and are defined in Z et al. this issue). Among the papers relevant for understanding the influence of OSIA on the aquatic environment, 95 did not include data to describe the Pressures of focus. Instead, many studies rely on other mechanisms, such as spatial proximity to establish relationships with oil sands development. Of the 28 papers which included data on Pressures (Supplemental table 2), these fall into two general categories: 24 included explicit data on all oil sands-related pressures (Supplemental table 2) while the remainder generally refers to an oil sands related Pressure. Among the oil-sands industry-related pressures, the two most common documented were emissions to the atmosphere (4 papers) and land disturbance (9 papers).

Among the 123 papers relevant to the state of the aquatic environment evaluated in the main manuscript, organic stressors, including polyaromatic compounds (PACs) and ‘naphthenic acids’ (NAs; also known as ‘naphthenic acid fraction compounds: NAFCs) were measured in 49 studies. Similarly, inorganic stressors (elements) were evaluated in 52. Other chemical or physical stressors, such as hydrology (12 papers), sediments (3 papers), and nutrients (14 papers) have received comparatively less attention in the peer-reviewed literature. The deposition of acidifying compounds to various landforms and their potential consequences were examined in 13 papers. Cumulative stresses were considered in 11 papers.

Pathways between OSIA include transport via the atmosphere, rivers, groundwater, or overland flow (Supplemental Figure 14). Among the published literature, measurements associated with atmospheric deposition (66 papers) and fluvial transport (57 papers) dominated the studies (Supplemental table 2). In many cases, these physical pathways were not documented with data and are instead inferred.

Among Responses, chemical changes in water quality and sediment quality were examined in 44 and 36 publications, respectively. Among organisms, the most commonly measured were fish (29 studies) and primary producers (18 papers). Measurements in fish, which include tissue concentrations of substances of concern, are the most common in the literature (Supplemental table 2; Evans et al. 2012; Evans et al. 2019; Tendler et al. 2020) but are accompanied by studies examining physiological change (Tetreault et al. 2020), and estimates of health (McMaster et al. 2020); Supplemental Table 1). Among the published literature, potential change in amphibians has only appeared in a single peer-reviewed publication (Mundy et al. 2019). Explicit linkages between results provided in papers and effects on humans (traditional use and human health) are considered in three papers.

# SUPPLEMENTAL LITERATURE REVIEWS

## ***Atmospheric deposition to snow***

Many studies have examined the occurrence of contaminants of concern (CoCs) in snow. Proximity to mines has been used to characterize spatial patterns of PACs and metals in the snow (Kirk et al. 2014; Manzano et al. 2016; Gopalapillai et al. 2019), and have been documented in lake sediments (Kurek et al. 2013) and wetlands (Wayland et al. 2008; Boutin and Carpenter 2017; Mundy et al. 2019). Among snow studies in the oil sands region, maximum deposition is predicted and has been observed within 50 km of a geographical reference point equidistant from the Syncrude and Suncor upgraders commonly known as AR6 (i.e., Kelly et al. 2009). This general spatial pattern centered on AR6 was also observed in bulk deposition (Bari et al. 2014) and additional surveys of the snowpack (Cho et al. 2014; Wang et al. 2014; Manzano et al. 2016; Chibwe et al. 2019), but has not been identified in naphthenic acid fraction compounds (Parrott et al. 2018).

Manzano et al. (2016) estimated 800-1,800 kg of PACs are deposited during the winter within 50 km of AR6. The deposition patterns have been attributed to the emissions from upgraders at the Suncor Base Mine and Syncrude Mildred Lake sites, but also suggest the contribution of both petrogenic and pyrogenic sources (Kelly et al. 2009). More recent evidence suggests petroleum coke may be the source of PACs in the snow (Chibwe et al. 2019) and other media (Zhang et al. 2016). Additional research in snow suggests PACs originating from OSIA may be transported up to 100 km north to the PAD (Manzano et al. 2017).

Similar to organic compounds, inorganic substances emitted from oil sands operations can accumulate in snow during the winter (Gopalapillai et al. 2019). Kelly et al. (2010) suggested four spatial patterns of the deposition were apparent for metals related to upgraders, local sources, and rates of analytical detection. Spatial pattern 1 was characterized as a steep exponential increase in maximum concentration with proximity to upgraders (Kelly et al. 2010) and was observed in particulate Be, Pb, Hg, and Ni (Kelly et al. 2010). Spatial pattern 2 suggests an influence of both emissions from upgrading facilities and local sources and was observed in dissolved Sb, Cr, Cu, Ni, Tl, and Zn and particulate Sb, As, Cd, Cr, Cu, Ag, Tl, and Zn (Kelly et al. 2010). Pattern 3 shows spatial uniformity and suggests local sources. While dissolved Cd, Pb, and Hg conform to this pattern of deposition (Kelly et al. 2010) it may also indicate the influence of deposition from global sources (e.g., Hope 2008; Rauch and Pacyna 2009; Selin 2009; Schlesinger et al. 2017). Undetectable metals, in particulate (Se) or dissolved form (As, Be, Se, Ag) were classified as spatial pattern 4 (Kelly et al. 2010).

Type 1 and 2 patterns have also been observed in other studies. Kirk et al. (2014) confirmed observations of Kelly et al. (2010) in Hg, Ni, Zn, but also found spatial patterns in Al, V, Fe, and La. Type 1 deposition patterns were also observed in 28 metals by Bari et al. (2014). Similar patterns are also apparent in total suspended sediments (TSS), supporting the role of particulates in either delivering and/or sequestering metals (Kirk et al. 2014). In contrast to Kelly et al. (2009) and Kirk et al. (2014), Bari et al. 2014) did not find strong spatial relationships between Hg and AR6. Additional results also conform to the hypothesized patterns of exponential decay for deposition with increasing distance from AR6, such as As, Ni, Cr, V, Mo, and S (Bari et al. 2014).

Much research has examined the potential sources of materials found in the environment around oil sands facilities (e.g., Gopalapillai et al. 2019). Studies of source materials demonstrate different compositions of minerals, including enrichment of Ni, V, and Zn in upgrader stack emissions relative to dust from haul roads, overburden, and processed materials (Landis et al. 2012). In contrast, Al, La, Fe, and Pb are enriched in haul road and overburden dust compared to emissions complexes used to upgrade bitumen to synthetic crude (Landis et al. 2012). Enrichment of metals in these various materials suggests their potential origin, but overlapping plumes from multiple sources of particulates and gases may also be responsible. Studies on snow support the roles of petroleum coke and road dust on metals in the snow (Gopalapillai et al. 2019). However, this work also reveals a general decrease in loading over time, similar to some metals in lake sediments, such as V (Cooke et al. 2017) suggesting improvements in the environmental performance of the industry and regional environmental health.

In contrast to the hypothesized sources of most lithophilic metals, identifying sources of Hg has been less straightforward (Willis et al. 2018). Upgraders and fugitive dust are commonly mentioned as potential sources of Hg in the AOSR (e.g., Kelly et al. 2010; Kirk et al. 2014; Willis et al. 2018) and research has suggested the Hg and MeHg is largely particulate-bound (Kirk et al. 2014; Willis et al. 2018). However, methyl-Hg (MeHg) occurs at low concentrations close to development when accounting for particle weight (0.53-6.85 ng/g; Willis et al. 2018). This suggests total particulate loading may be responsible for the patterns generated through spatial interpolation in earlier work (Kirk et al. 2014). As discussed in the main text, the role of changes in facility-specific practices over time may more specifically explain differences observed in studies from different years.

Other factors may also influence patterns in the snow. While the role of topography in the deposition patterns has not been specifically examined, other work suggests a potential role in the deposition patterns observed (Biagi and Carey 2020). Topography may account for some of the additional structure apparent in spatial regressions of contaminants, such as the increase in variability as samples are collected closer to AR6 (i.e., Manzano et al. 2016). Annual variability may also influence these data as can the attributes of particles transporting substances of concern as identified in studies examining different indicators (Landis et al. 2019). More detailed analyses which account for known and extraneous sources of variation may be necessary.

Estimating the potential toxicological harm of substances deposited in environmental media is done using several techniques, including the comparison to environmental quality guidelines (e.g., Kelly et al. 2010). Melted snow and water from the Athabasca River and tributaries contained concentrations of Cd, Cu, Pb, Hg, Ni, Ag, and Zn above the Canadian Council of Ministers of the Environment (CCME) water quality guidelines for the protection of aquatic life, but not drinking water quality guidelines (Kelly et al. 2010). Similarly, exceedances were observed in melted snow in Pb, Zn, Fe, and Al in 2012 (Kirk et al. 2014). In contrast, in all snow samples from 2011-2012 total Hg, Ni, MeHg were below the CCME water quality guidelines for the protection of aquatic life (Kirk et al. 2014). Similarly, metals were below CCME water quality guidelines for the protection of aquatic life in ion-amended snows used for laboratory exposures (Parrott et al. 2018).

While acidifying compounds are associated with potential effects on environmental pH, the compounds can also cause nutrient enrichment. Nutrients in snow have been reported in a single study examined in this review (Summers et al. 2016). Using snow samples collected in March 2014, Summers et al. (2016) reported patterns in total nitrogen and total phosphorus similar to those reported for metals (Kirk et al. 2014). In contrast, dissolved inorganic nitrogen (DIN), total dissolved phosphorus (TDP), and soluble reactive phosphorus (SRP) appear more diffuse suggesting multiple sources, slow deposition velocities, both, or another factor (Summers et al. 2016).

## ***Atmospheric deposition to wetlands***

Similar to snow, the atmospheric deposition of substances of concern has been evaluated in some wetlands (Shotyk et al. 2014). Ombrotrophic bogs have been used to examine atmospheric deposition in the oil sands region. Data from the rain-fed bogs demonstrated strong associations between multiple metals and a conservative tracer, Th (Shotyk et al. 2014; Donner et al. 2019) indicating the delivery of contaminants via mineral dust (Landis et al. 2012) echoing the results of other studies (X et al. this issue; (Landis et al. 2012; Shotyk et al. 2015; Shotyk, Bicalho, et al. 2016; Cooke et al. 2017; Landis et al. 2019). Particle sizes and dust types may, however, also explain additional variation in some metals such as V (Shotyk et al. 2014; Mullan-Boudreau et al. 2017). Mullan-Boudreau et al. (2017) suggest multiple sources of dust in the oil sands region are apparent, similar to Landis et al. (2012, 2019a), Shotyk et al. (2014; 2016), Stachiw et al. (2019), and Gopalapillai et al. (2019).

Similar to data from lake sediments (Cooke et al. 2017), peat cores, suggest many metals, not just Pb and V, have been declining in the OSR for decades (Shotyk, Appleby, et al. 2016; Shotyk et al. 2017). Boutin and Carpenter (2017) suggest higher metals are found in soils of wetlands and upland vegetation closer to oil sands facilities, but Shotyk (2018) suggests these observations may be better explained by spatial differences in soil type and natural mineral content, rather than proximity to the development. This criticism highlights the common occurrence of natural gradients accompanying the results of some studies which may not be ruled out. Later work also found some metals on the surface of berries collected from bogs delivered via dust (Al, Cr, Pb, U, and V) whereas others are absorbed from soils (Ba, Cd, Cu, Mn, Mo, Ni, Rb, Sr, Zn; Stachiw et al. 2019). Additional work suggests no spatial gradients in Hg are observed in mosses collected in the OSR (Shotyk and Cuss 2019).

The potential for nutrient enrichment in bogs sensitive to nutrifying compounds, including N emitted from oil sands operations, and the prevalence of this wetland type in the OSR has motivated research on this topic (Wieder et al. 2010; Wieder, Vile, Albright, et al. 2016; Wieder, Vile, Scott, et al. 2016). Bogs reflect direct wet and dry deposition of compounds via the atmosphere (Wieder et al. 2010). Deposition of S and N may also affect biological processes (Vile et al. 2014) and biogeochemical cycling (Wieder et al. 2010; Wieder et al. 2019), with alterations possibly concentration-dependent (Wieder et al. 2010; Stuart et al. 2018; Wieder et al. 2019).

Studies of potential effects of passive N and S deposition in bogs has been ongoing in the OSR since at least 2005. In this work, no discernible patterns were found in N or S deposition in bogs from 2005-2008 (Wieder et al. 2010). However, later work shows results corresponding to those from lake sediments (Cooke et al. 2017), snow (Kelly et al. 2009), lichens (Landis et al. 2012), and soils (Watmough et al. 2014), where deposition of N (NO_3_^-^), S (SO_4_^2-^), Ca^2+^, and Mg^2+^ in bogs was above background within 20 km of the Syncrude and Suncor upgraders (Wieder, Vile, Albright, et al. 2016); Figure 3) In contrast to other deposition studies, this recent work suggests signals of DIN and SO_4_^2-^ in bogs may extend 250 km (Wieder, Vile, Albright, et al. 2016), however, no sites between 100 and 249 km from the geographical reference point were sampled. Research on ombrotrophic bogs showed there was not an exponential decline with distance from the geographical reference point (~AR6) in ortho-phosphate (Wieder, Vile, Albright, et al. 2016). A diffuse pattern was also observed in Acid Soluble P (Mullan-Boudreau et al. 2017) suggesting multiple sources of P in the OSR paralleling earlier results in snow (Summers et al. 2016)

Few studies examining physiological or community health of ambient wetlands were identified during this review, however, some are available and generally include ombrotrophic bogs (e.g., Wieder et al. 2010). Similar to bulk deposition of N and S, tissue concentrations of these elements increased with proximity to AR6 in some photosynthetic organisms (Wieder, Vile, Scott, et al. 2016).

Polyaromatic compounds have also been examined in organisms residing in wetlands. In contrast to the spatial patterns of chemicals measured in vegetation, the occurrence of PACs in insect larvae was not spatially-dependent (Wayland et al. 2008). A similar result (not spatially dependent) was also found in wood frog tadpoles collected from wetlands. In contrast to data from semi-permeable membrane devices (SPMDs), PACs measured in the tissues of tadpoles did not vary spatially (Mundy et al. 2019). These data suggest wood frogs are exposed to organic contaminants but are depurating the compounds, similar to inferences from sentinel fish analyses (Tetreault et al. 2003,2020; McMaster et al. 2018a, b; McMaster et al. 2020).

The potential influence of elemental deposition originating from OSIA on wetland vegetation has also been examined. Growth rates of *Sphagnum fuscum* did not differ among bogs sampled at varying distances from the mid-point between the Syncrude and Suncor upgraders in a study from 2005-2008 (Wieder et al. 2010). However, a later study (2009-2014) found growth of *S. fuscum* was highest at a site within 20 km of a point midway between the Syncrude and Suncor upgraders, but was also more variable and not correlated with NH_4_^+^-N, NO_3_^-^-N, or DIN deposition (Wieder, Vile, Albright, et al. 2016). Furthermore, more species have been found in sites closer to OS development (Boutin and Carpenter 2017). These differences have largely been attributed to the occurrence of more non-native species closer to OSIA (Boutin and Carpenter 2017). Similar results were also reported for soil seedbanks (Boutin and Carpenter 2017). Finally, throughout the administrative boundaries of the Athabasca, Peace, and Cold Lake regions, local disturbances of all human development were apparent in vegetation communities of wetlands (Ficken et al. 2019).

## ***Status of the Peace Athabasca Delta***

Changing water levels in the Peace Athabasca Delta (See Figure 1 of main text) have been observed and can affect resident organisms (Ward and Gorelick 2018; Bush et al. 2020) but the causes are contested (e.g., Beltaos 2018; Hall et al. 2018; Timoney et al. 2019). Although researchers (Wolfe et al. 2008; Wolfe et al. 2012) suggest flooding cycles have been predominantly influenced by climate variability, others have suggested contrasting conclusions (e.g., Beltaos 2018). Associations with water withdrawals for oil sands mining have not been demonstrated in the PAD, but the high natural variability in the benthic communities, reflecting the annual, seasonal, and daily fluctuations inherent in deltas and flowing water systems has recently appeared (Bush et al. 2020).

Substances of concern occur in the PAD (Timoney and Lee 2009), a culturally significant area. These compounds may originate from OSIA or natural processes. The PAD is a low-relief area and promotes the settling of materials, but is also subject to high spatial and temporal variability (Bush et al. 2020). Particles in the PAD may also be delivered through atmospheric or fluvial mechanisms. For example, PACs can be eroded from the McMurray Formation in tributaries and banks of the mainstem and delivered to depositional areas downstream (Akre et al. 2004; Droppo et al. 2019), including the PAD; Figure 1)

*Polyaromatic compounds*

A potential association between oil sands development influencing the mobilization and occurrence of PACs in downstream environments and its potential change over time has motivated studies in flood-prone lakes of the PAD and Slave River Delta (SRD). Data from surface sediments collected from locations in distributaries show patterns suggesting an increase in PAH between 1999 and 2014 (Evans et al. 2016), 1999-2009 (Timoney and Lee 2011), and mean aPAC concentration measured between 1999 and 2007 (Timoney and Lee 2009). In contrast, Hall et al. (2012) suggest there has been no detectable change in anthropogenic PACs inputs to flood-prone lakes in the PAD since the beginning of oil sands mining and the prevailing role of natural processes (Kay et al. 2020; Owca et al. 2020). Jautzy et al. (2015) also present data from PAD23 suggesting PAHs and aPACs have been declining over time. Differences in the conclusions of these studies may be associated with different sampling techniques and materials collected, such as river bed sediments compared to deposited sediments in flood-prone lakes (Hall et al. 2012). Sampling in the SRD 500 km downstream of the oil sands area shows no discernible signals of oil sands development and no clear evidence of any monotonic changes over time (Elmes et al. 2016). Similarly, PAD15, a lake north of Lake Athabasca in the PAD shows evidence of petrogenic PAHs which may derive from coal and bitumen in the Peace River watershed (Jautzy, Ahad, Hall, et al. 2015). Similar to lakes in NW Saskatchewan which may be sensitive to long-range transport of OS-derived substances (Ahad et al. 2015), PAD15 also shows evidence of pyrogenic PAHs likely attributable to forest fires (Jautzy, Ahad, Hall, et al. 2015). Irrespective of the source, the concentration of parent and alkyl-PACs in the sediments of PAD15 declined over time (Jautzy, Ahad, Hall, et al. 2015).

*Metals and trace elements*

Metals have been measured in aquatic media in the PAD and reported in the peer-reviewed literature. Dissolved As in water was higher at three locations (near the water intake for Fort Chipewyan, Rochers River near Mission Creek, and Fletcher Channel) in the PAD in 2007 (1.6-3.4 μg/L) compared to their median values calculated using data from 1976-87 (~0.6 mg/L; Timoney and Lee 2009 and references therein). Sediments collected from PAD distributaries and near the Fort Chipewyan water intake in 2000 were also 35-114% of medians calculated using data from 1976-1999 (Timoney and Lee 2009 and references therein). However, no clear upstream to downstream spatial patterns were found in As in the Athabasca River in 2014 or 2015 (Donner et al. 2017).

Sediments have also been collected in the PAD lakes and rivers. Arsenic in sediments from near the Fort Chipewyan water intake and in the Rochers River were 9.2 and 9.1 mg/kg, respectively in 2007 (Timoney and Lee 2009 and references therein). Wiklund et al. (2014) also examined the change in metals in delta distributaries. Using sediments deposited in floodplain lakes, expected concentrations of metals relative to Li were calculated. Using these expected ranges, Wiklund et al. (2014) concluded most Li-normalized metal concentrations in the PAD and distributaries of the Athabasca River (Be, Cr, Pb, C, Zn, Ni, Cu, and Cd) were delivered to two PAD lakes (PAD 23 and 31) from 2010-2013 were not different than expected from the 1700-1900 reference period. While residuals for some metals were occasionally above the upper prediction limit, such as Cu, these potential increases were transient but may require further attention and reporting. Although the metals generally showed little unexpected variance, these researchers did not evaluate As (Wiklund et al. 2014). More recent research supports these findings (Kay et al. 2020).

Sediments of floodplain lakes and distributaries in the PAD and SRD have also been examined by researchers. No patterns in metals in the SRD appear associated with oil sands development based on the onset of mining (MacDonald et al. 2016). Similarly, As, Pb, Sb, and Hg in PAD18, a perched lake, has declined over time (Wiklund et al. 2012), suggesting a decline in atmospheric inputs over time. Similar to PACs measured in flood-prone lakes, river transport may be the most likely route of transport to the PAD (Hall et al. 2012).

*Biotic effects*

The potential impacts of oil sands development on biota in the PAD, including natural resources are a common concern (Kelly et al. 2009; Timoney and Lee 2009; Schindler 2013). Some analyses of fish tissues for chemicals of concern have also been done in the PAD. (Evans and Talbot 2012) found mercury concentrations decreased over time in western Lake Athabasca and the PAD in northern pike (1981–2009), but not in walleye (1981–2005) or lake trout (1978–2009). Tendler et al. (2020) also collected fish near Fort Chipewyan. These researchers found Hg in the tissues of walleye were often above consumption and subsistence guidelines (Tendler et al. 2020).

Few studies have examined changes in organisms in the PAD. However, recent work suggests the predominant role of spatial and temporal variability in the diversity of benthic communities (Bush et al. 2020).

Supplemental References

Akre CJ, Headley J V., Conly FM, Peru KM, Dickson LC. 2004. Spatial Patterns of Natural Polycyclic Aromatic Hydrocarbons in Sediment in the Lower Athabasca River. J Environ Sci Heal - Part A Toxic/Hazardous Subst Environ Eng. 39(5):1163–1176. doi:10.1081/ESE-120030301.

Bari M a., Kindzierski WB, Cho S. 2014. A wintertime investigation of atmospheric deposition of metals and polycyclic aromatic hydrocarbons in the Athabasca Oil Sands Region, Canada. Sci Total Environ. 485–486(1):180–192. doi:10.1016/j.scitotenv.2014.03.088.

Beltaos S. 2018. Frequency of ice-jam flooding of peace-athabasca delta. Can J Civ Eng. 45(1):71–75. doi:10.1139/cjce-2017-0434.

Biagi KM, Carey SK. 2020. The role of snow processes and hillslopes on runoff generation in present and future climates in a recently constructed watershed in the Athabasca oil sands region. Hydrol Process. 34(17):3635–3655. doi:10.1002/hyp.13836.

Blais JM, Donahue WF. 2015. Comment on “Sphagnum mosses from 21 ombrotrophic bogs in the Athabasca bituminous sands region show no significant atmospheric contamination of ‘heavy metals.’” Environ Sci Technol. 49(10):6352–6353.

Boutin C, Carpenter DJ. 2017. Assessment of wetland/upland vegetation communities and evaluation of soil-plant contamination by polycyclic aromatic hydrocarbons and trace metals in regions near oil sands mining in Alberta. Sci Total Environ. 576:829–839. doi:10.1016/j.scitotenv.2016.10.062.

Bush A, Monk WA, Compson ZG, Peters DL, Porter TM, Shokralla S, Wright MTG, Hajibabaei M, Baird DJ. 2020. DNA metabarcoding reveals metacommunity dynamics in a threatened boreal wetland wilderness. Proc Natl Acad Sci U S A. 117(15):8539–8545. doi:10.1073/pnas.1918741117.

Chibwe L, Manzano CA, Muir D, Atkinson B, Kirk JL, Marvin CH, Wang X, Teixeira C, Shang D, Harner T, et al. 2019. Deposition and Source Identification of Nitrogen Heterocyclic Polycyclic Aromatic Compounds in Snow, Sediment, and Air Samples from the Athabasca Oil Sands Region. Environ Sci Technol. 53(6):2981–2989. doi:10.1021/acs.est.8b06175.

Cho S, Sharma K, Brassard BW, Hazewinkel R. 2014. Polycyclic aromatic hydrocarbon deposition in the snowpack of the Athabasca oil sands region of Alberta, Canada. Water Air Soil Pollut. 225(5). doi:10.1007/s11270-014-1910-4.

Cooke C a., Kirk JL, Muir DCG, Wiklund J a., Wang X, Gleason A, Evans MS. 2017. Spatial and temporal patterns in trace element deposition to lakes in the Athabasca oil sands region (Alberta, Canada). Environ Res Lett. 12(12). doi:10.1088/1748-9326/aa9505.

Donner MW, Bicalho B, Noernberg T, Shotyk W. 2019. Contemporary and Historical Atmospheric Deposition of Arsenic and Selenium in the Athabasca Bituminous Sands Region. Environ Sci Technol. 53(23):14020–14028. doi:10.1021/acs.est.9b02718.

Donner MW, Javed MB, Shotyk W, Francesconi KA, Siddique T. 2017. Arsenic speciation in the lower Athabasca River watershed: A geochemical investigation of the dissolved and particulate phases. Environ Pollut. 224:265–274. doi:10.1016/j.envpol.2017.02.004. http://dx.doi.org/10.1016/j.envpol.2017.02.004.

Droppo IG, di Cenzo P, Parrott J, Power J. 2019. The Alberta oil sands eroded bitumen/sediment transitional journey: Influence on sediment transport dynamics, PAH signatures and toxicological effect. Sci Total Environ. 677:718–731. doi:10.1016/j.scitotenv.2019.04.313. https://doi.org/10.1016/j.scitotenv.2019.04.313.

Elmes MC, Wiklund JA, Van Opstal SR, Wolfe BB, Hall RI. 2016. Characterizing baseline concentrations, proportions, and processes controlling deposition of river-transported bitumen-associated polycyclic aromatic compounds at a floodplain lake (Slave River Delta, Northwest Territories, Canada). Environ Monit Assess. 188(5):1–15. doi:10.1007/s10661-016-5277-4.

Evans M, Davies M, Janzen K, Muir D, Hazewinkel R, Kirk J, De Boer D. 2016. PAH distributions in sediments in the oil sands monitoring area and western Lake Athabasca: Concentration, composition and diagnostic ratios. Environ Pollut. 213:671–687. doi:10.1016/j.envpol.2016.03.014.

Evans MS, McMaster M, Muir DCG, Parrott J, Tetreault GR, Keating J. 2019. Forage fish and polycyclic aromatic compounds in the Fort McMurray oil sands area: Body burden comparisons with environmental distributions and consumption guidelines. Environ Pollut. 255:113135. doi:10.1016/j.envpol.2019.113135. https://doi.org/10.1016/j.envpol.2019.113135.

Evans MS, Talbot A. 2012. Investigations of mercury concentrations in walleye and other fish in the Athabasca River ecosystem with increasing oil sands developments. J Environ Monit. 14(7):1989–2003. doi:10.1039/c2em30132f.

Evans MS, Talbot A, j.jhydrol.2016.11.034. Evans MS, Talbot A. 2012. Investigations of mercury concentrations in walleye and other fish in the Athabasca River ecosystem with increasing oil sands developments. J Environ Monit. 14(7):1989–2003. doi:10.1039/c2em30132f.

Ficken CD, Cobbaert D, Rooney RC. 2019. Low extent but high impact of human land use on wetland flora across the boreal oil sands region. Sci Total Environ. 693:133647.

Gopalapillai Y, Kirk JL, Landis MS, Muir DCG, Cooke CA, Gleason A, Ho A, Kelly E, Schindler D, Wang X, et al. 2019. Source Analysis of Pollutant Elements in Winter Air Deposition in the Athabasca Oil Sands Region: A Temporal and Spatial Study. ACS Earth Sp Chem. 3(8):1656–1668. doi:10.1021/acsearthspacechem.9b00150.

Hall RI, Wolfe BB, Wiklund J a., Edwards TWD, Farwell AJ, Dixon DG. 2012. Has Alberta Oil Sands Development Altered Delivery of Polycyclic Aromatic Compounds to the Peace-Athabasca Delta? PLoS One. 7(9). doi:10.1371/journal.pone.0046089.

Hall RI, Wolfe BB, Wiklund JA. 2018. Discussion of “Frequency of ice-jam flooding of Peace-Athabasca Delta.” Can J Civ Eng. 46(3):236–238.

Jautzy J, Ahad JME, Gobeil C, Savard MM. 2013. Century-long source apportionment of PAHs in athabasca oil sands region lakes using diagnostic ratios and compound-specific carbon isotope signatures. Environ Sci Technol. 47(12):6155–6163. doi:10.1021/es400642e.

Jautzy JJ, Ahad JME, Gobeil C, Smirnoff A, Barst BD, Savard MM. 2015. Isotopic Evidence for Oil Sands Petroleum Coke in the Peace-Athabasca Delta. Environ Sci Technol. 49(20):12062–12070. doi:10.1021/acs.est.5b03232.

Jautzy JJ, Ahad JME, Hall RI, Wiklund JA, Wolfe BB, Gobeil C, Savard MM. 2015. Source Apportionment of Background PAHs in the Peace-Athabasca Delta (Alberta, Canada) Using Molecular Level Radiocarbon Analysis. Environ Sci Technol. 49(15):9056–9063. doi:10.1021/acs.est.5b01490.

Kay ML, Wiklund JA, Remmer CR, Owca TJ, Klemt WH, Neary LK, Brown K, MacDonald E, Thomson K, Vucic JM, et al. 2020. Evaluating temporal patterns of metals concentrations in floodplain lakes of the Athabasca Delta (Canada) relative to pre-industrial baselines. Sci Total Environ. 704:135309. doi:10.1016/j.scitotenv.2019.135309. https://doi.org/10.1016/j.scitotenv.2019.135309.

Kelly EN, Schindler DW, Hodson P V., Short JW, Radmanovich R, Nielsen CC. 2010. Oil sands development contributes elements toxic at low concentrations to the Athabasca River and its tributaries. Proc Natl Acad Sci. 107(37):16178–16183. doi:10.1073/pnas.1008754107.

Kelly EN, Short JW, Schindler DW, Hodson P V, Ma M, Kwan AK, Fortin BL. 2009. Oil sands development contributes polycyclic aromatic compounds to the Athabasca River and its tributaries. Proc Natl Acad Sci. 106(52):22346–22351.

Kirk JL, Muir DCGG, Gleason A, Wang X, Lawson G, Frank RA, Lehnherr I, Wrona F. 2014. Atmospheric deposition of mercury and methylmercury to landscapes and waterbodies of the athabasca oil sands region. Environ Sci Technol. 48(13):7374–7383. doi:10.1021/es500986r.

Korosi JB, Irvine G, Skierszkan EK, Doyle JR, Kimpe LE, Janvier J, Blais JM. 2013. Localized enrichment of polycyclic aromatic hydrocarbons in soil, spruce needles, and lake sediments linked to in-situ bitumen extraction near Cold Lake, Alberta. Environ Pollut. 182:307–315. doi:10.1016/j.envpol.2013.07.012.

Kurek J, Kirk JL, Muir DCGG, Wang X, Evans MS, Smol JP. 2013. Legacy of a half century of Athabasca oil sands development recorded by lake ecosystems. Proc Natl Acad Sci. 110(5):1761–1766. doi:10.1073/pnas.1217675110.

Landis MS, Pancras JP, Graney JR, Stevens RK, Percy KE, Krupa S. 2012. Receptor Modeling of Epiphytic Lichens to Elucidate the Sources and Spatial Distribution of Inorganic Air Pollution in the Athabasca Oil Sands Region. Dev Environ Sci. 11:427–467. doi:10.1016/B978-0-08-097760-7.00018-4.

Landis MS, Studabaker WB, Patrick Pancras J, Graney JR, Puckett K, White EM, Edgerton ES. 2019. Source apportionment of an epiphytic lichen biomonitor to elucidate the sources and spatial distribution of polycyclic aromatic hydrocarbons in the Athabasca Oil Sands Region, Alberta, Canada. Sci Total Environ. 654(1241–1257):1241–1257. doi:10.1016/j.scitotenv.2018.11.131. https://doi.org/10.1016/j.scitotenv.2018.11.131.

MacDonald L a., Wiklund J a., Elmes MC, Wolfe BB, Hall RI. 2016. Paleolimnological assessment of riverine and atmospheric pathways and sources of metal deposition at a floodplain lake (Slave River Delta, Northwest Territories, Canada). Sci Total Environ. 544:811–823. doi:10.1016/j.scitotenv.2015.11.173.

Manzano CA, Muir D, Kirk J, Teixeira C, Siu M, Wang X, Charland JP, Schindler D, Kelly E, Zhang YF. 2016. Temporal variation in the deposition of polycyclic aromatic compounds in snow in the Athabasca Oil Sands area of Alberta. Environ Monit Assess. 188(9):10–1007. doi:10.1007/s10661-016-5500-3.

McMaster ME, Tetreault GR, Clark T, Bennett J, Cunningham J, Ussery EJ, Evans M. 2020. Baseline white sucker health and reproductive endpoints for use in assessment of further development in the alberta oil sands. Int J Environ Impacts Manag Mitig Recover. 3(3):219–237. doi:10.2495/ei-v3-n3-219-237.

Mullan-Boudreau G, Belland R, Devito K, Noernberg T, Pelletier R, Shotyk W. 2017. Sphagnum Moss as an Indicator of Contemporary Rates of Atmospheric Dust Deposition in the Athabasca Bituminous Sands Region. Environ Sci Technol. 51(13):7422–7431. doi:10.1021/acs.est.6b06195.

Mundy LJ, Bilodeau JC, Schock DM, Thomas PJ, Blais JM, Pauli BD. 2019. Using wood frog (*Lithobates sylvaticus*) tadpoles and semipermeable membrane devices to monitor polycyclic aromatic compounds in boreal wetlands in the oil sands region of northern Alberta, Canada. Chemosphere. 214:148–157. doi:10.1016/j.chemosphere.2018.09.034. https://doi.org/10.1016/j.chemosphere.2018.09.034.

Owca TJ, Kay ML, Faber J, Remmer CR, Zabel N, Wiklund JA, Wolfe BB, Hall RI. 2020. Use of pre-industrial baselines to monitor anthropogenic enrichment of metals concentrations in recently deposited sediment of floodplain lakes in the Peace-Athabasca Delta (Alberta, Canada). Environ Monit Assess. 192(2). doi:10.1007/s10661-020-8067-y.

Parrott JL, Marentette JR, Hewitt LM, McMaster ME, Gillis PL, Norwood WP, Kirk JL, Peru KM, Headley J V., Wang Z, et al. 2018. Meltwater from snow contaminated by oil sands emissions is toxic to larval fish, but not spring river water. Sci Total Environ. 625:264–274. doi:10.1016/j.scitotenv.2017.12.284.

Schindler DW. 2013. Geoscience of Climate and Energy 12. Water quality issues in the oil sands region of the Lower Athabasca River, Alberta. Geosci Canada. 40(3):202–214.

Shotyk W. 2018. Trace metals in soils of the bituminous sands mining region of Alberta: A critical, geochemical perspective on the study by Boutin and Carpenter (2017). Sci Total Environ. 618:866–869. doi:10.1016/j.scitotenv.2017.08.233. https://doi.org/10.1016/j.scitotenv.2017.08.233.

Shotyk W, Appleby PG, Bicalho B, Davies L, Froese D, Grant-Weaver I, Krachler M, Magnan G, Mullan-Boudreau G, Noernberg T, et al. 2016. Peat bogs in northern Alberta, Canada reveal decades of declining atmospheric Pb contamination. Geophys Res Lett. 43(18):9964–9974. doi:10.1002/2016GL070952.

Shotyk W, Appleby PG, Bicalho B, Davies LJ, Froese D, Grant-Weaver I, Magnan G, Mullan-Boudreau G, Noernberg T, Pelletier R, et al. 2017. Peat Bogs Document Decades of Declining Atmospheric Contamination by Trace Metals in the Athabasca Bituminous Sands Region. Environ Sci Technol. 51(11):6237–6249. doi:10.1021/acs.est.6b04909.

Shotyk W, Belland R, Duke J, Kempter H, Krachler M, Noernberg T, Pelletier R, Vile MA, Wieder K, Zaccone C. 2014. Sphagnum mosses from 21 ombrotrophic bogs in the Athabasca bituminous sands region show no significant atmospheric contamination of “heavy metals.” Environ Sci Technol. 48(21):12603–12611.

Shotyk W, Belland R, Noernberg T, Pelletier R, Zaccone C, Zhang S, Duke J, Kempter H, Krachler M, Vile M a., et al. 2015. Response to Comment on “sphagnum mosses from 21 ombrotrophic bogs in the athabasca bituminous sands region show no significant atmospheric contamination of ‘heavy metals.’” Environ Sci Technol. 49(10):6354–6357. doi:10.1021/acs.est.5b01229.

Shotyk W, Bicalho B, Cuss CW, Duke MJM, Noernberg T, Pelletier R, Steinnes E, Zaccone C. 2016. Dust is the dominant source of “heavy metals” to peat moss (Sphagnum fuscum) in the bogs of the Athabasca Bituminous Sands region of northern Alberta. Environ Int. 92–93:494–506. doi:10.1016/j.envint.2016.03.018.

Shotyk W, Cuss CW. 2019. Atmospheric Hg accumulation rates determined using Sphagnum moss from ombrotrophic (rain-fed) bogs in the Athabasca Bituminous Sands region of northern Alberta, Canada. Ecol Indic. 107(January):105626. doi:10.1016/j.ecolind.2019.105626. https://doi.org/10.1016/j.ecolind.2019.105626.

Stachiw S, Bicalho B, Grant-Weaver I, Noernberg T, Shotyk W. 2019. Trace elements in berries collected near upgraders and open pit mines in the Athabasca Bituminous Sands Region (ABSR): Distinguishing atmospheric dust deposition from plant uptake. Sci Total Environ. 670:849–864. doi:10.1016/j.scitotenv.2019.03.238.

Stuart JEM, Wieder RK, Vile M a. 2018. Net nitrogen mineralization in Alberta bog peat is insensitive to experimentally increased nitrogen deposition and time since wildfire. Biogeochemistry. 138(2):155–170. doi:10.1007/s10533-018-0437-y.

Summers JC, Kurek J, Kirk JL, Muir DCG, Wang X, Wiklund JA, Cooke CA, Evans MS, Smol JP. 2016. Recent Warming , Rather than Industrial Emissions of Bioavailable Nutrients , Is the Dominant Driver of Lake Primary Production Shifts across the Athabasca Oil Sands Region. PLoS One. 11(5):1–20. doi:10.5061/dryad.k37q7.

Tendler B, Ohiozebau E, Codling G, Giesy JP, Jones PD. 2020. Concentrations of Metals in Fishes from the Athabasca and Slave Rivers of Northern Canada. Environ Toxicol Chem. 00(00):1–16. doi:10.1002/etc.4852.

Tetreault GR, Bennett CJ, Clark TW, Keith H, Parrott JL, McMaster ME. 2020. Fish Performance Indicators Adjacent to Oil Sands Activity: Response in Performance Indicators of Slimy Sculpin in the Steepbank River, Alberta, Adjacent to Oil Sands Mining Activity. Environ Toxicol Chem. 39(2):396–409.

Timoney K, Smith JD, Lamontagne JR, Jasek M. 2019. Discussion of “Frequency of ice-jam flooding of Peace-Athabasca Delta.” Can J Civ Eng. 46(3):239–242.

Timoney KP, Lee P. 2009. Does the Alberta Tar Sands Industry Pollute? The Scientific Evidence. Open Conserv Biol J. 3(1):65–81. doi:10.2174/1874839200903010065.

Timoney KP, Lee P. 2011. Polycyclic aromatic hydrocarbons increase in athabasca river delta sediment: Temporal trends and environmental correlates. In: Environmental Science and Technology. Vol. 45. p. 4278–4284.

Vile M a., Kelman Wieder R, Živković T, Scott KD, Vitt DH, Hartsock J a., Iosue CL, Quinn JC, Petix M, Fillingim HM, et al. 2014. N2-fixation by methanotrophs sustains carbon and nitrogen accumulation in pristine peatlands. Biogeochemistry. 121(2):317–328. doi:10.1007/s10533-014-0019-6.

Wang Z, Yang C, Parrott JL, Frank RA, Yang Z, Brown CE, Hollebone BP, Landriault M, Fieldhouse B, Liu Y, et al. 2014. Forensic source differentiation of petrogenic, pyrogenic, and biogenic hydrocarbons in Canadian oil sands environmental samples. J Hazard Mater. 271:166–177. doi:10.1016/j.jhazmat.2014.02.021. http://dx.doi.org/10.1016/j.jhazmat.2014.02.021.

Ward EM, Gorelick SM. 2018. Drying drives decline in muskrat population in the Peace-Athabasca Delta, Canada. Environ Res Lett. 13(12):124026.

Watmough SA, Whitfield CJ, Fenn ME. 2014. The importance of atmospheric base cation deposition for preventing soil acidification in the Athabasca Oil Sands Region of Canada. Sci Total Environ. 493:1–11. doi:10.1016/j.scitotenv.2014.05.110.

Wayland M, Headley J V., Peru KM, Crosley R, Brownlee BG. 2008. Levels of polycyclic aromatic hydrocarbons and dibenzothiophenes in wetland sediments and aquatic insects in the oil sands area of Northeastern Alberta, Canada. Environ Monit Assess. 136(1–3):167–182. doi:10.1007/s10661-007-9673-7.

Wieder RK, Vile MA, Albright CM, Scott KD, Vitt DH, Quinn JC, Burke-Scoll M. 2016. Effects of altered atmospheric nutrient deposition from Alberta oil sands development on Sphagnum fuscum growth and C, N and S accumulation in peat. Biogeochemistry. 129(1–2):1–19. doi:10.1007/s10533-016-0216-6.

Wieder RK, Vile MA, Scott KD, Albright CM, McMillen KJ, Vitt DH, Fenn ME. 2016. Differential Effects of High Atmospheric N and S Deposition on Bog Plant/Lichen Tissue and Porewater Chemistry across the Athabasca Oil Sands Region. Environ Sci Technol. 50(23):12630–12640. doi:10.1021/acs.est.6b03109.

Wieder RK, Vitt DH, Burke-Scoll M, Scott KD, House M, Vile M a. 2010. Nitrogen and sulphur deposition and the growth of Sphagnum fuscum in bogs of the Athabasca Oil Sands Region, Alberta. J Limnol. 69(SUPPL. 1):161–170. doi:10.3274/JL10-69-S1-16.

Wieder RK, Vitt DH, Vile M a., Graham J a., Hartsock J a., Fillingim H, House M, Quinn JC, Scott KD, Petix M, et al. 2019. Experimental nitrogen addition alters structure and function of a boreal bog: critical load and thresholds revealed. Ecol Monogr. 0(0):1–35. doi:10.1002/ecm.1371.

Wiklund J a., Hall RI, Wolfe BB, Edwards TWD, Farwell AJ, Dixon DG. 2012. Has Alberta oil sands development increased far-field delivery of airborne contaminants to the Peace-Athabasca Delta? Sci Total Environ. 433:379–382. doi:10.1016/j.scitotenv.2012.06.074.

Wiklund JA, Hall RI, Wolfe BB, Edwards TWD, Farwell AJ, Dixon DG. 2014. Use of pre-industrial floodplain lake sediments to establish baseline river metal concentrations downstream of Alberta oil sands: a new approach for detecting pollution of rivers. Environ Res Lett. 9(12):124019.

Willis CE, Kirk JL, St Louis VL, Lehnherr I, Ariya PA, Rangel-Alvarado RB. 2018. Sources of Methylmercury to Snowpacks of the Alberta Oil Sands Region: A Study of in Situ Methylation and Particulates. Environ Sci Technol. 52(2):531–540. doi:10.1021/acs.est.7b04096.

Wolfe BB, Hall RI, Edwards TWD, Johnston JW. 2012. Developing temporal hydroecological perspectives to inform stewardship of a northern floodplain landscape subject to multiple stressors: paleolimnological investigations of the Peace–Athabasca Delta. Environ Rev. 20(3):191–210. doi:10.1139/a2012-008.

Wolfe BB, Hall RI, Edwards TWD, Vardy SR, Falcone MD, Sjunneskog C, Sylvestre F, McGowan S, Leavitt PR, van Driel P. 2008. Hydroecological responses of the Athabasca Delta, Canada, to changes in river flow and climate during the 20th century. Ecohydrol Ecosyst L Water Process Interact Ecohydrogeomorphology. 1(2):131–148.

# SUPPLEMENTAL TABLES

Supplemental table 1 Start up dates and production capacity of Oil Sands Facilities; data obtained from Oil Sands Magazine (<https://www.oilsandsmagazine.com/projects/bitumen-production>) [Accessed November 15, 2020.] ; N/A=not available; BPD=barrels per day.

| **Operator** | **Facility** | **Type** | **Start-up year** | **Production capacity (10^3^ BPD)** | **Notes** |
| --- | --- | --- | --- | --- | --- |
| Suncor | Basemine | Mine | 1967 | N/A |  |
| Imperial | Cold Lake | In situ | 1975/1985 | N/A | pilot/commercial |
| Syncrude | Mildred Lake | In situ | 1978 | 150 |  |
| Canadian Natural | Wolflake/Primrose/Burnt Lake | In situ | 1985 | 120 |  |
| Canadian Natural | Peace River/Carmon Creek | In situ | 1986 | 12.5 |  |
| Greenfire | Hangingstone Demo | In situ | 1999 | 10 |  |
| Cenovus | Foster Creek | In situ | 2001 | 180 |  |
| Suncor | Millenium | Mine | 2001 | 180 |  |
| Syncrude | Aurora North | In situ | 2001 | 225 |  |
| Canadian Natural | Muskeg River | Mine | 2002 | 175 |  |
| Cenovus | Christina Lake | In situ | 2002 | 260 |  |
| Suncor | Mackay River | In situ | 2002 | 38 | pilot in 1983 |
| Suncor | Firebag | In situ | 2004 | 215 |  |
| Husky | Tucker Lake | In situ | 2006 | 30 |  |
| ConocoPhillips | Surmont | In situ | 2007 | 148 |  |
| OSUM | Orion | In situ | 2007 | 18 |  |
| CNOOC | Nexen | In situ | 2008 | 92 |  |
| Connacher | Great Divide | In situ | 2008 | 20 |  |
| MEG | Christina Lake | In situ | 2008 | 100 |  |
| Canadian Natural | Horizon | Mine | 2009 | 294 |  |
| Athabasca | Leismer | In situ | 2010 | 20 |  |
| Canadian Natural | Jackpine | Mine | 2010 | 145 |  |
| Suncor | (North) Steepbank | Mine | 2010 | 150 |  |
| International Petroleum Corporation | Blackrod | In situ | 2011 | 0.5 |  |
| CONA | Lindbergh | In situ | 2012/2015 | N/A | pilot/commercial |
| Canadian Natural | Kirby | In situ | 2013 | 80 |  |
| Imperial | Kearl | Mine | 2013 | 220 |  |
| Athabasca | Hangingstone | In situ | 2015 | 12 |  |
| Husky | Sunrise | In situ | 2015 | 60 |  |
| Sunhsine | West Ells | In situ | 2015 | 5 |  |
| JACOS | Hangingstone EXP | In situ | 2017 | 20 |  |
| Petrochina | MacKay River | In situ | 2017 | 35 |  |
| Harvest | Blackgold | In situ | 2018 | 10 |  |
| Suncor | Fort Hills | Mine | 2018 | 194 |  |

Supplemental table 2 Number of studies examining specific biological measurements in organisms among the papers identified for this study.

| **Biological response** | **Organism** | | | | |
| --- | --- | --- | --- | --- | --- |
|  | **Amphibians** | **Fish** | **Invertebrate** | **Primary**  **producers** | **Protists** |
| Chlorophyll *a* |  |  |  | 3 |  |
| Community |  |  | 7 | 13 | 1 |
| Detoxification enzymes |  | 3 | 1 |  |  |
| Gene expression |  | 2 |  |  |  |
| Growth |  |  |  | 4 |  |
| Health |  | 7 |  |  |  |
| Larval survival |  | 1 |  |  |  |
| Parasite community |  | 2 |  |  |  |
| Physical abnormalities |  | 4 |  |  |  |
| Spawning populations |  | 2 |  |  |  |
| Stable isotopes |  | 2 |  |  |  |
| Tissue burdens | 1 | 18 | 2 |  |  |
| Tolerance |  |  |  | 1 |  |
| Size |  | 1 |  |  |  |
| Relative abundance |  | 1 |  |  |  |

# SUPPLEMENTAL FIGURES


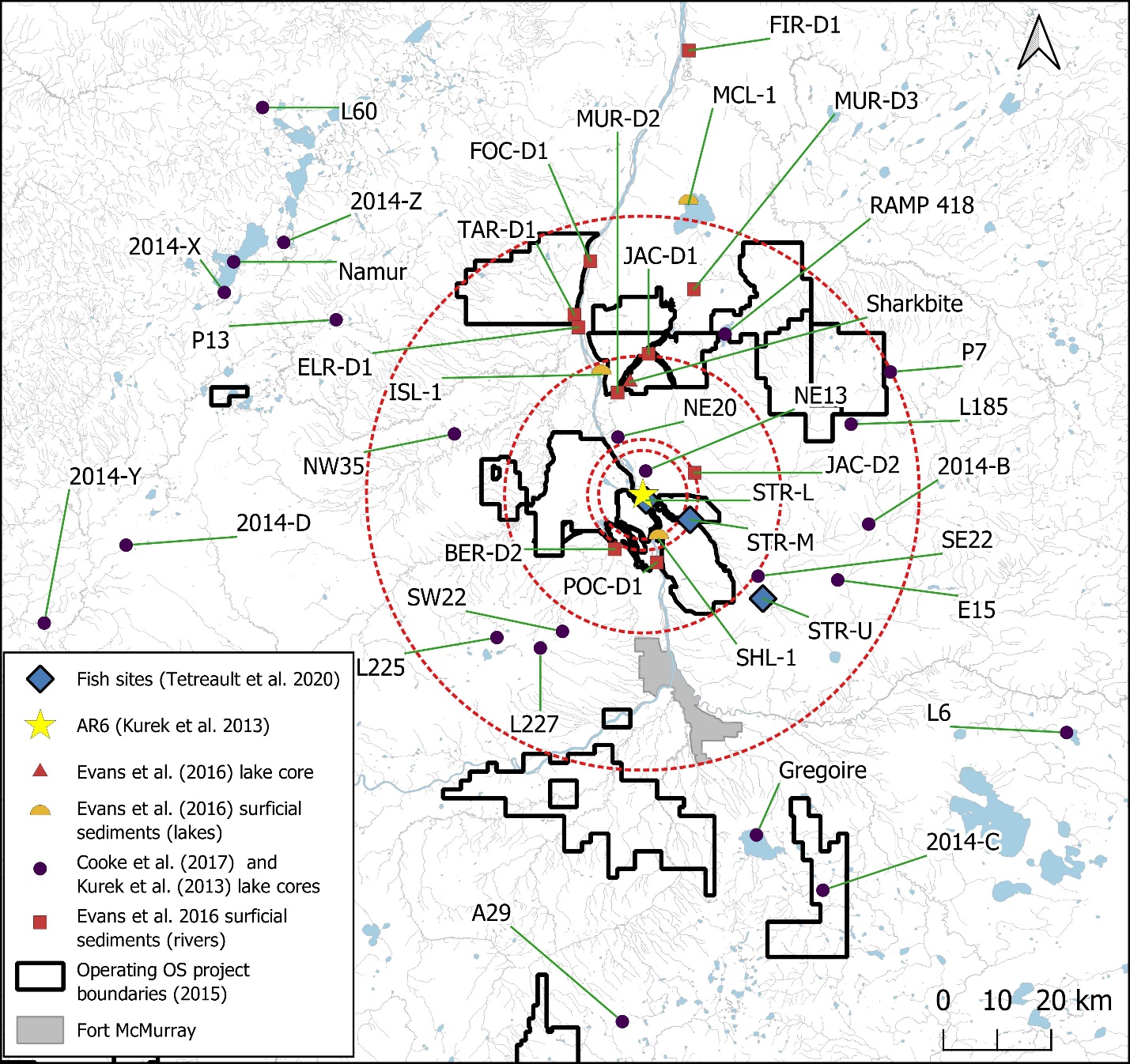


Supplemental Figure 1 Sampling locations used in the peer-reviewed literature including lakes cores, surficial sediments from lakes and rivers, fishing locations, AR6 and AR6 distance bands (8, 10, 25, and 50 km); sediment core also collected by Evans et al. (2016) at RAMP 418 (Kearl Lake); details of industrial infrastructure omitted for clarity; project boundaries of facilities operating in 2015 shown.


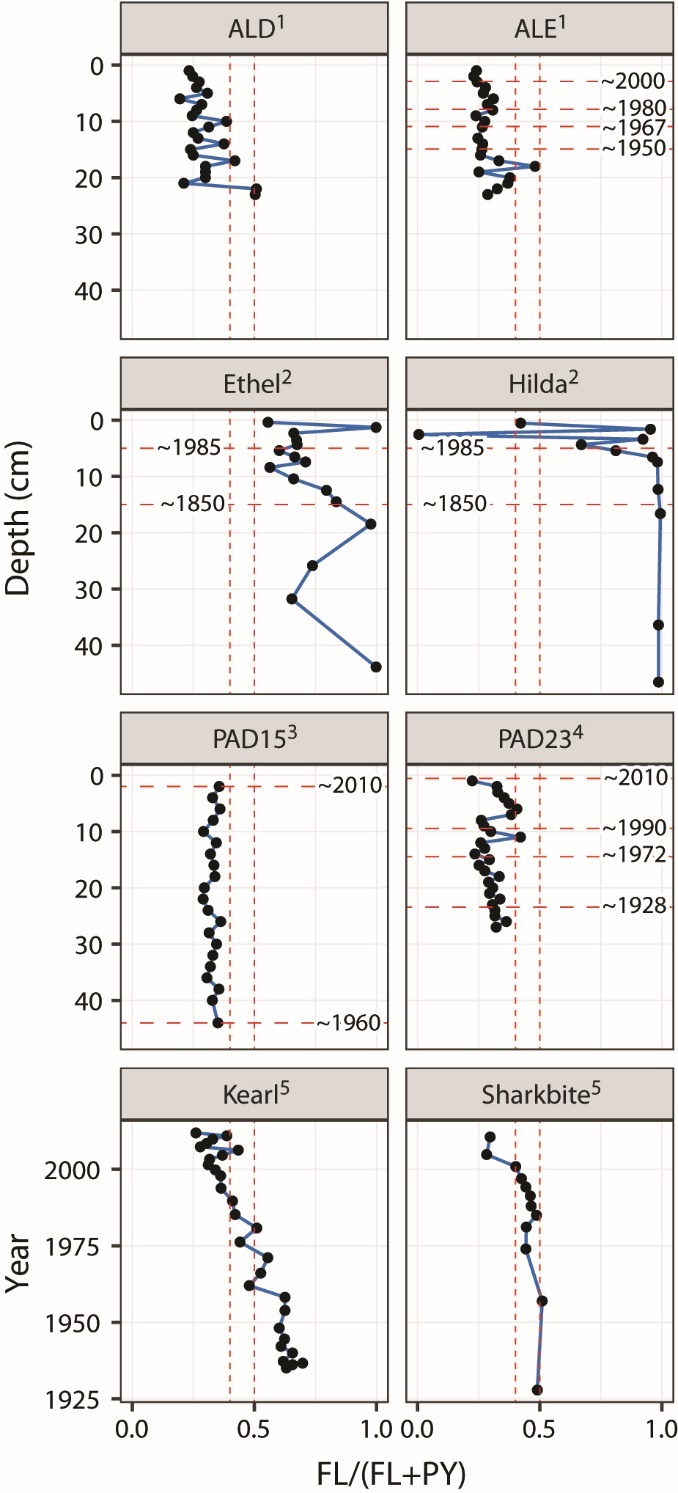


Supplemental Figure 2 Ratios of fluoranthene to fluoranthene plus pyrene in lake sediment cores from the eastern Athabasca Oil Sands Region (ALD and ALE), the Cold Lake Region (Ethel and Hilda), the Peace Athabasca Delta (PAD15 and PAD23), and the minable region (Kearl and Sharkbite); Dates not provided for ALD core in source material; ^1^(Jautzy et al. 2013); ^2^(Korosi et al. 2013); ^3^(Jautzy, Ahad, Hall, et al. 2015); ^4^(Jautzy, Ahad, Gobeil, et al. 2015); ^5^(Evans et al. 2016); vertical dashed lines denote petrogenic (<0.4), petroleum combustion (0.4<x<0.5, pyrogenic (>0.5) signatures of FL/(FL+PY) ratio from literature (e.g., Yunker et al. 2002).


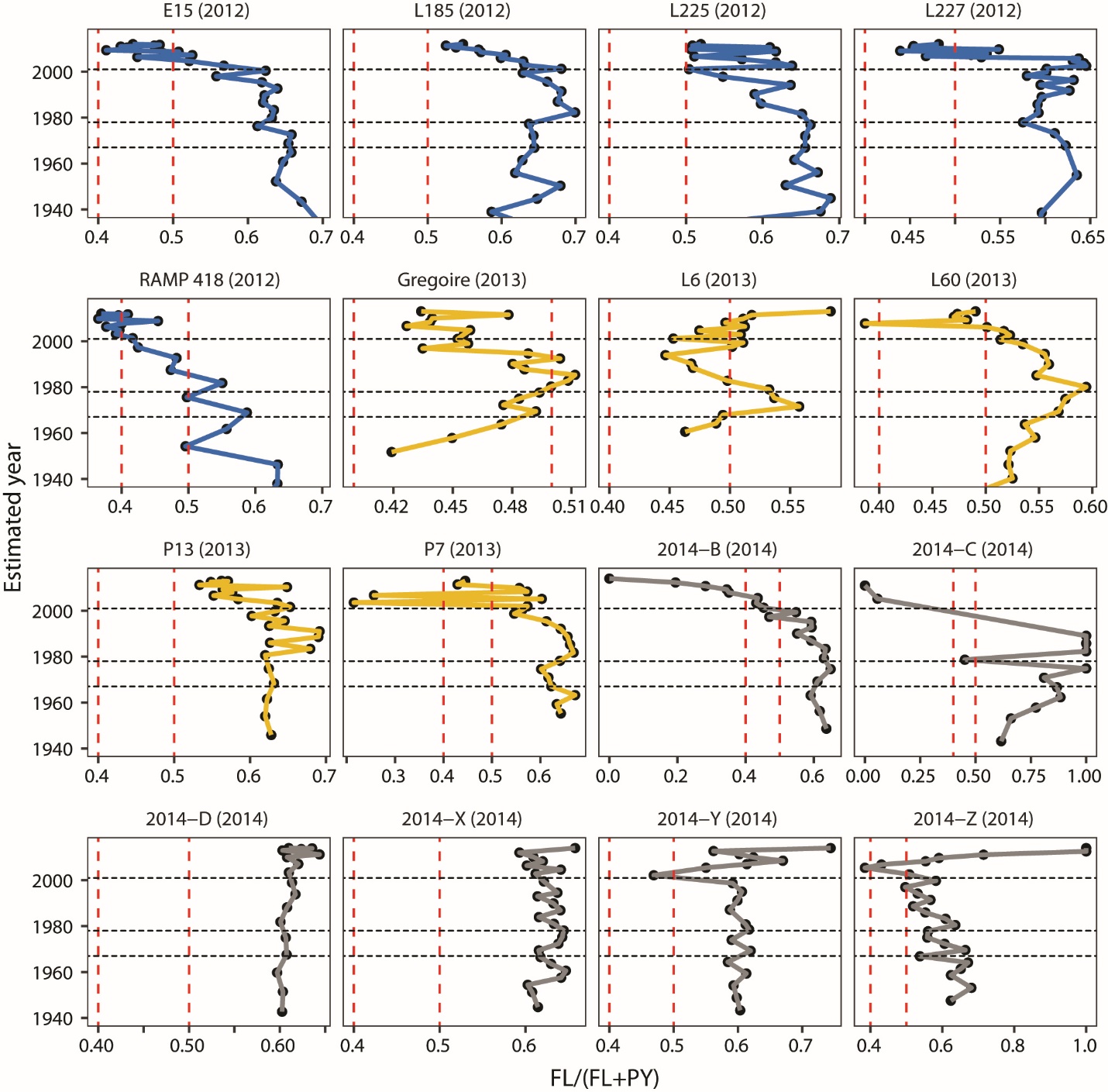


Supplemental Figure 3 Ratios of fluoranthene to fluoranthene plus pyrene (FL/(FL+PY)) in lake sediments compared to estimated years; horizontal dashed lines show commercial start-ups of Great Canadian Oil Sands (1967), Syncrude (1978), and Aurora North (2001); vertical dashed lines denote petrogenic (<0.4), petroleum combustion (0.4<x<0.5, pyrogenic (>0.5) signatures of FL/(FL+PY) ratio from literature (e.g., Yunker et al. 2002); data available from: <https://donnees.ec.gc.ca/data/substances/monitor/sediment-oil-sands-region/sediment-cores-paleo-analyses-oil-sands-region/>; dating of sediment cores available from Summers et al. (2016).


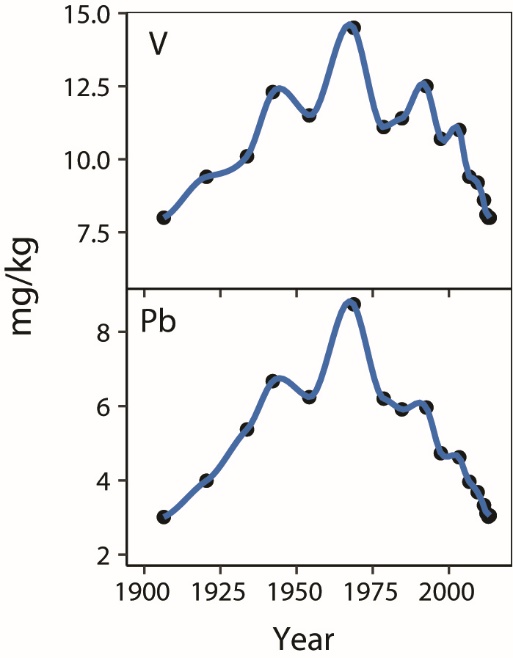


Supplemental Figure 4 Sediment core concentration (mg/kg) of vanadium (V) and lead (Pb) from Kearl Lake (also called Kearle and RAMP-418) sampled for the Oil Sands Monitoring program; data available from: <https://donnees.ec.gc.ca/data/substances/monitor/sediment-oil-sands-region/sediment-cores-paleo-analyses-oil-sands-region/>; dating of sediment cores available from Summers et al. (2016).


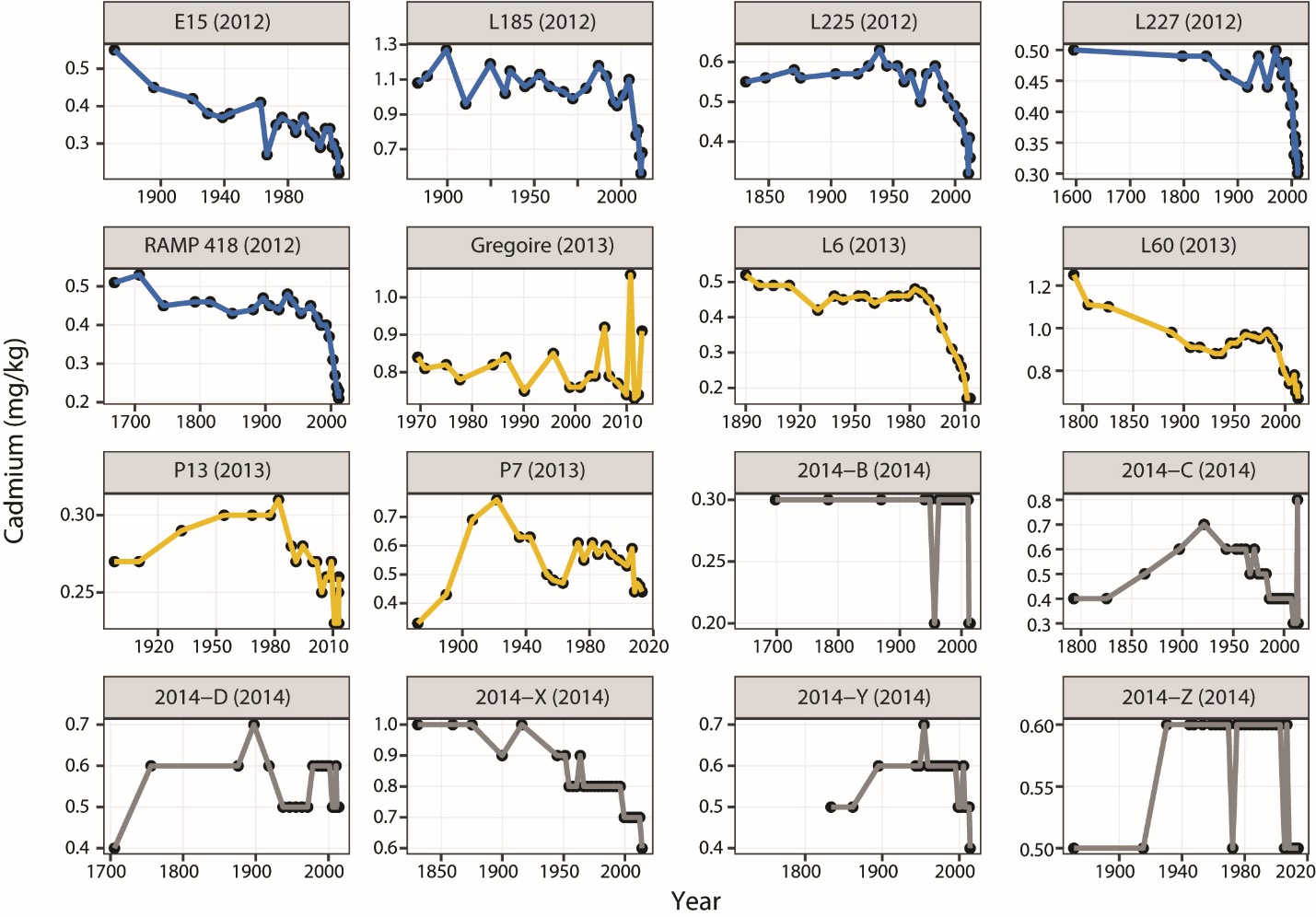


Supplemental Figure 5 Concentration of cadmium (mg/kg) from lakes sampled by Cooke et al. (2017) and Summers et al. (2016) relative to estimated deposition time showing both potential declines in Cd concentration over time (e.g., 2012- RAMP 418) and insufficient analytical precision in samples collected in 2014; colours indicate sampling years; data available from: <https://donnees.ec.gc.ca/data/substances/monitor/sediment-oil-sands-region/sediment-cores-paleo-analyses-oil-sands-region/>; dating of sediment cores available from Summers et al. (2016).


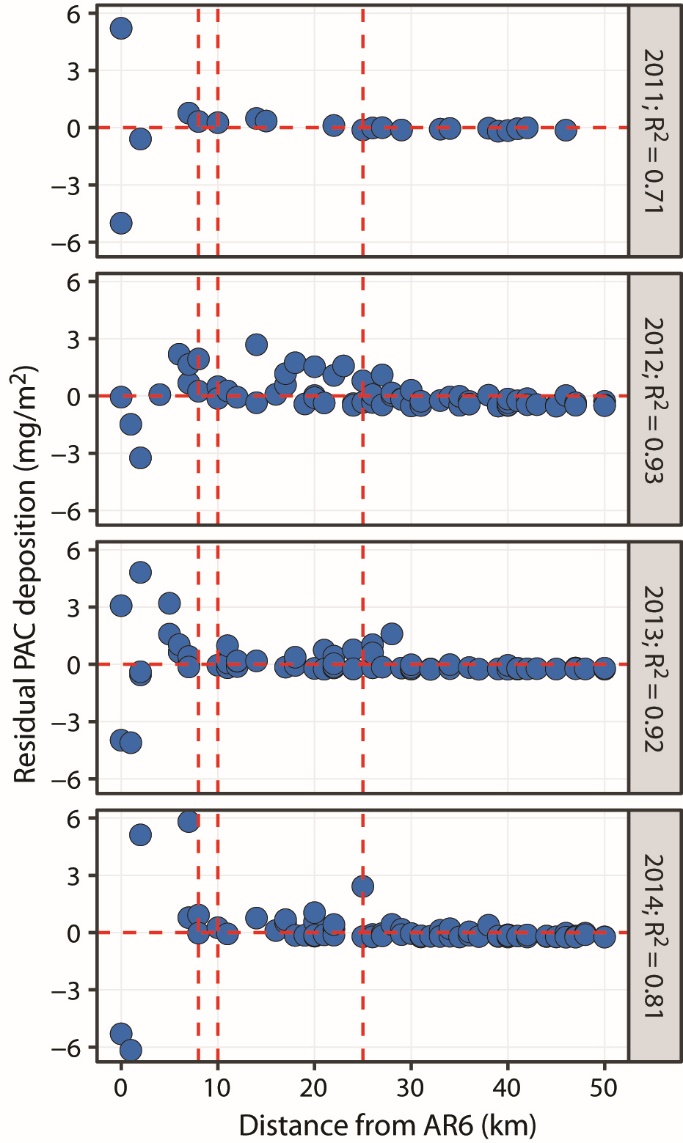


Supplemental Figure 6 Residuals of PACs deposited in snow versus distance from AR6 available from Manzano et al. (2016); vertical dashed lines mark 8, 10, and 25 km from AR6; horizontal dashed line marks 0.


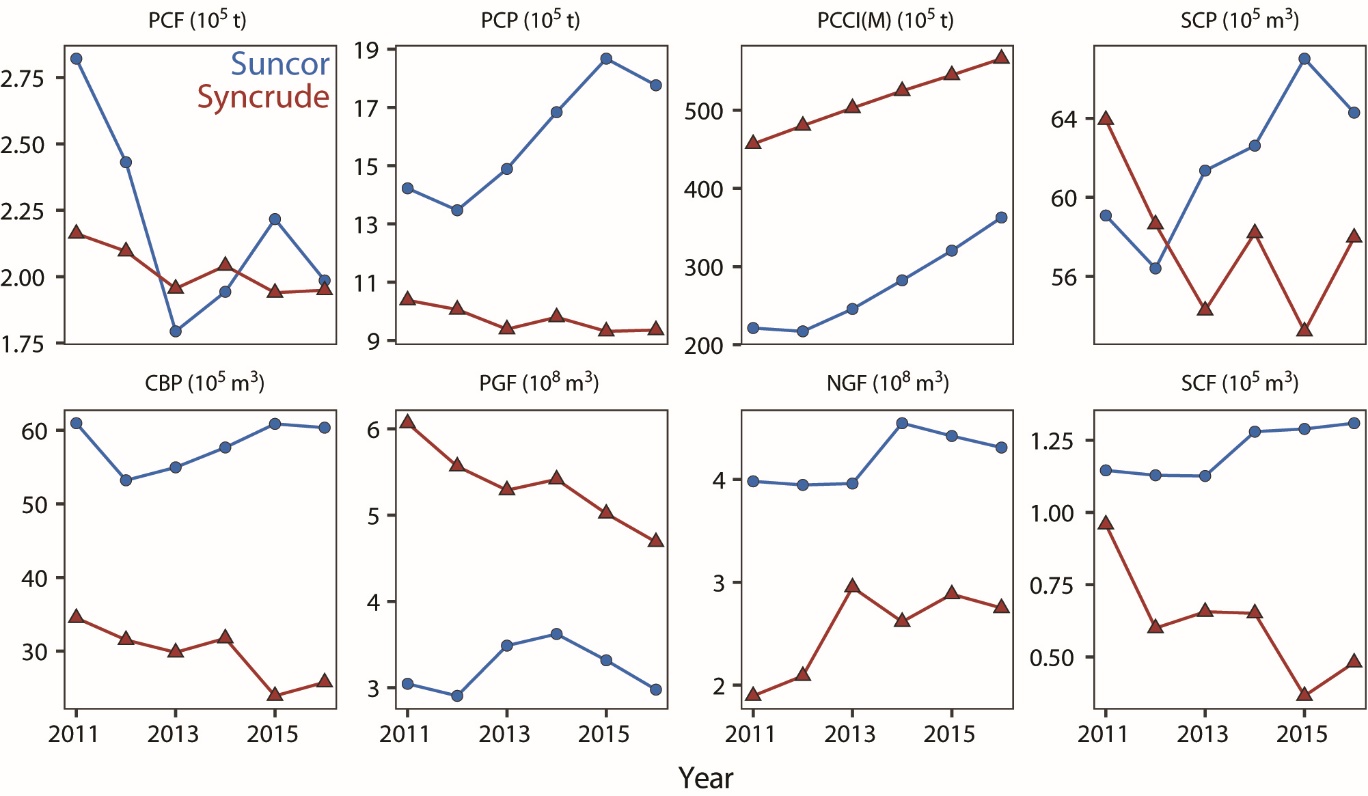


Supplemental Figure 7 Winter (December (Year_t-1_) and January-March(Year_t_)) production and fuel data at the Suncor Basemine and Syncrude Mildred Lake available from the Alberta Energy Regulator ST39 reports (<https://www.aer.ca/providing-information/data-and-reports/statistical-reports/st39>); PCF = petroleum coke as fuel; PCP = petroleum coke production; PCCI(M) = petroleum coke closing inventory in March; SCP = Synthetic crude production; CBP = crude bitumen production; PGF = process gas as fuel; NGF = natural gas as fuel; SCF=synthetic crude as fuel.


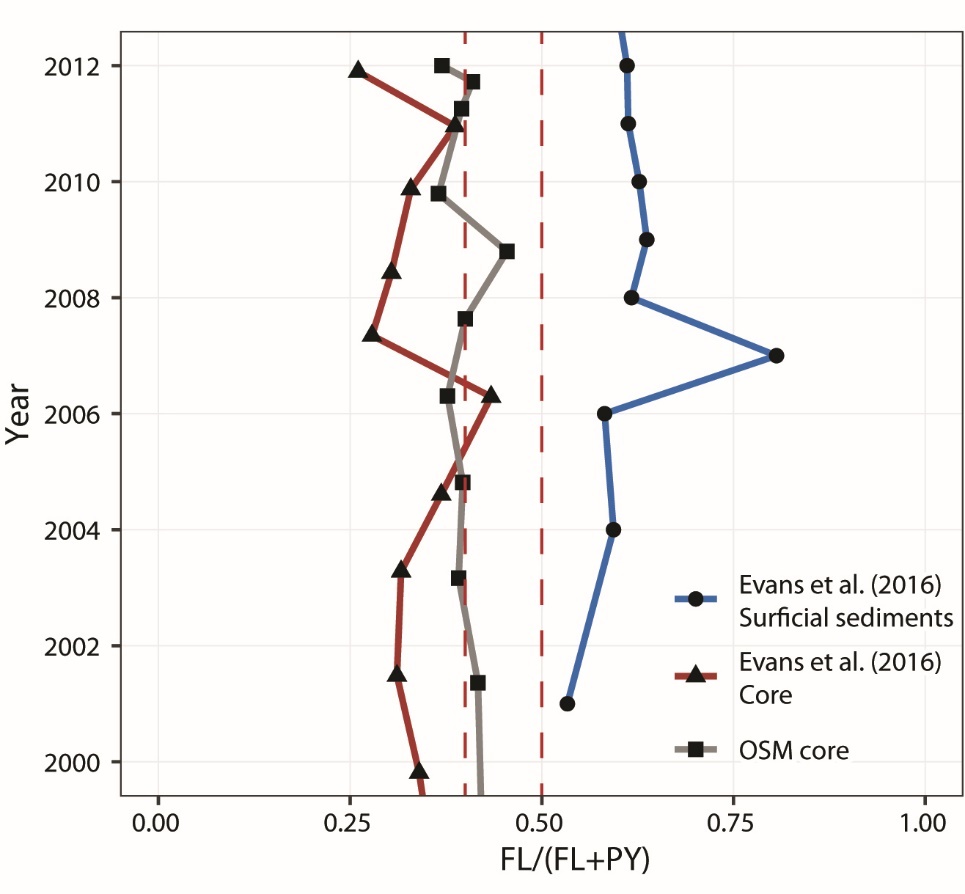


Supplemental Figure 8 Ratios of fluoranthene to fluoranthene plus pyrene (FL/(FL+PY)) in surficial sediments and sediment core of Kearl (also called Kearle and RAMP-418) available from Evans et al. (2016), Ramp-alberta.org, and the Oil Sands Monitoring (OSM) program (<https://www.canada.ca/en/environment-climate-change/services/oil-sands-monitoring/monitoring-water-quality-alberta-oil-sands.html>) from ~2000-2012, the period in which data from all three sources overlaps.


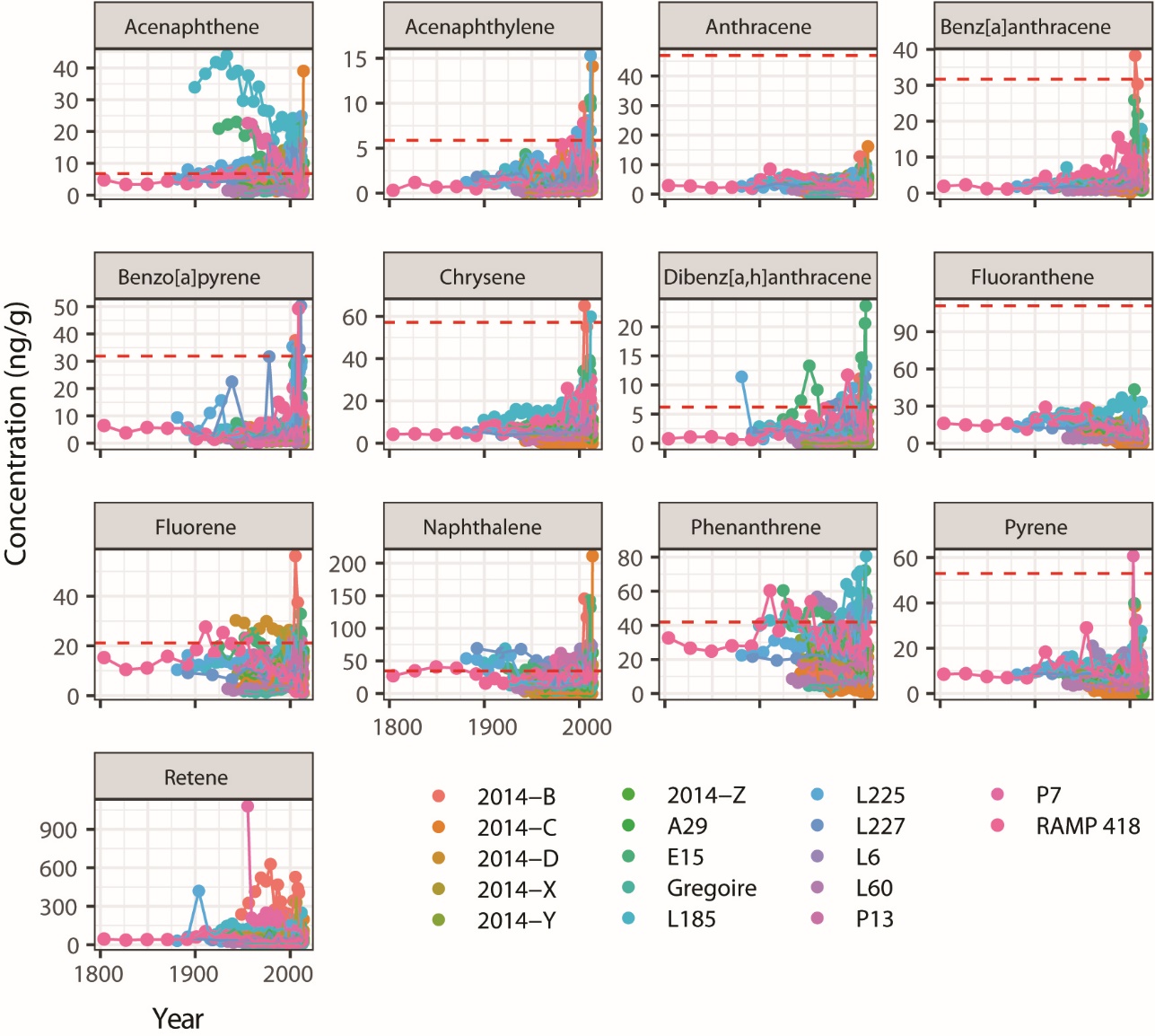


Supplemental Figure 9 Concentrations of PAHs (ng/g) from lakes sampled in the OSR (i.e., Summers et al. 2016) compared to Interim Sediment Quality Guidelines (horizontal dashed lines) over time; also includes Retene to account for potential influence of forest fires; locations of lakes shown in Supplemental Figure 1; area burned in wildfires in Alberta also shown in Supplemental Figure 10; data available from <https://www.canada.ca/en/environment-climate-change/services/oil-sands-monitoring/monitoring-water-quality-alberta-oil-sands.html>.


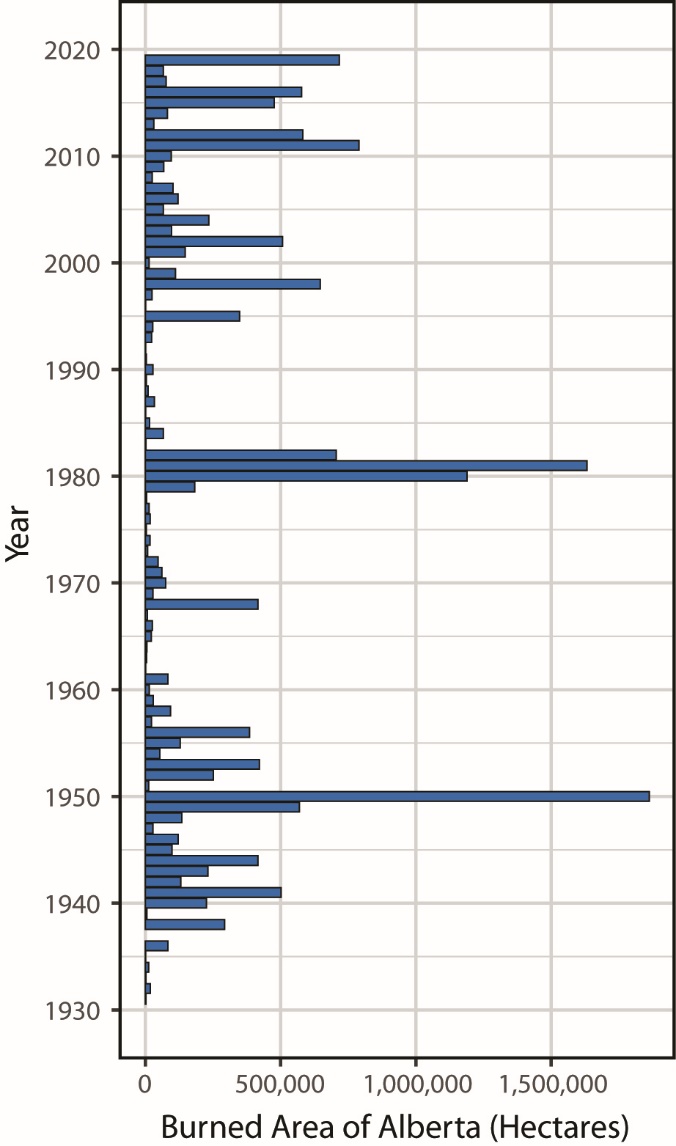


Supplemental Figure 10 Total area (Hectares) burned in Alberta by year from 1930-2019; Area calculated from shape file obtained: <https://wildfire.alberta.ca/resources/historical-data/spatial-wildfire-data.aspx> [Accessed June 2, 2021].


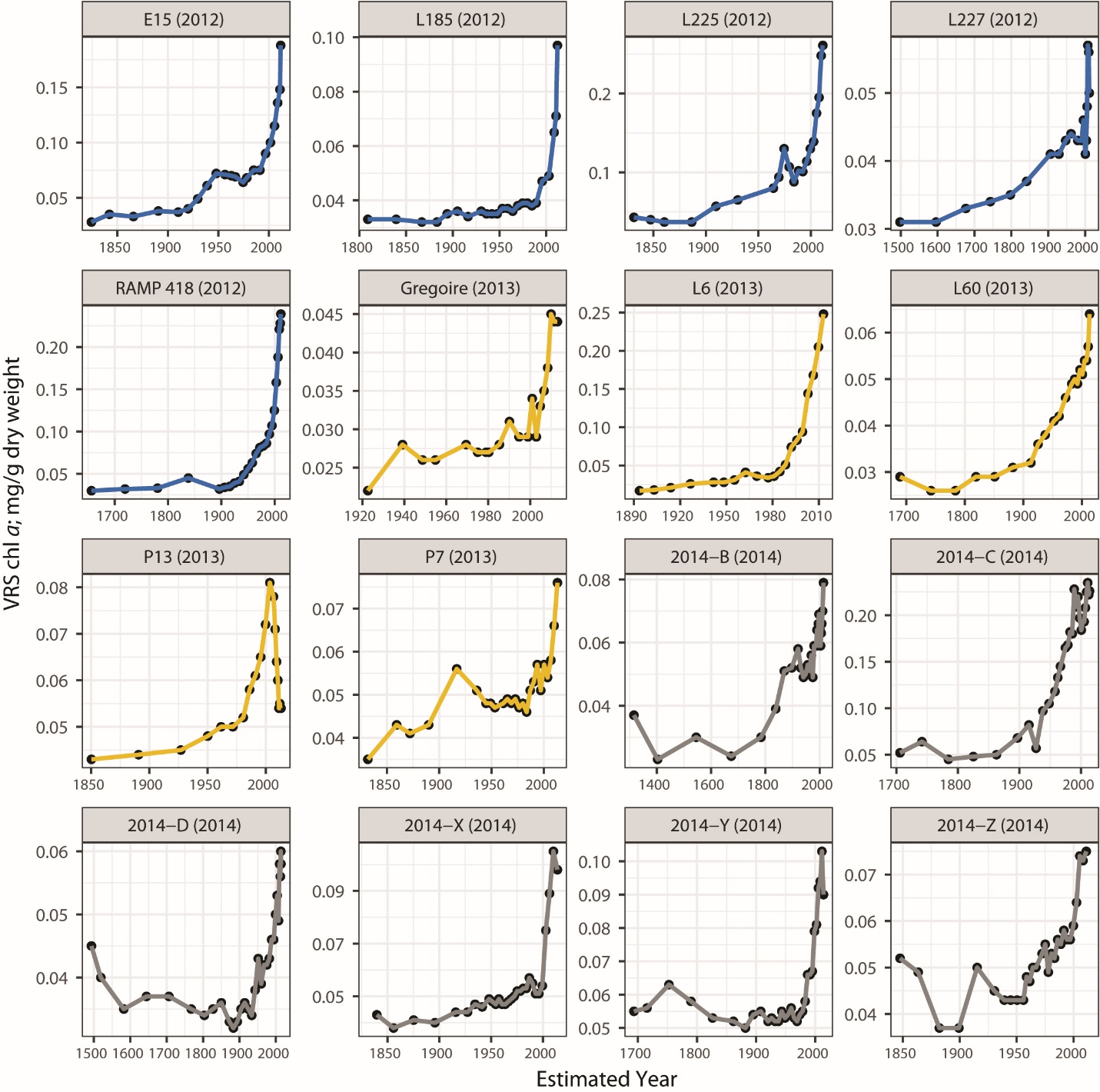


Supplemental Figure 11 Concentrations of Chlorophyll a in lake sediment sections estimated inferences using visible reflectance spectroscopy (VRS chl a; mg/g dry weight) from Summers et al. 2016 and available from <https://www.canada.ca/en/environment-climate-change/services/oil-sands-monitoring/monitoring-water-quality-alberta-oil-sands.html>; colours indicate sampling years.


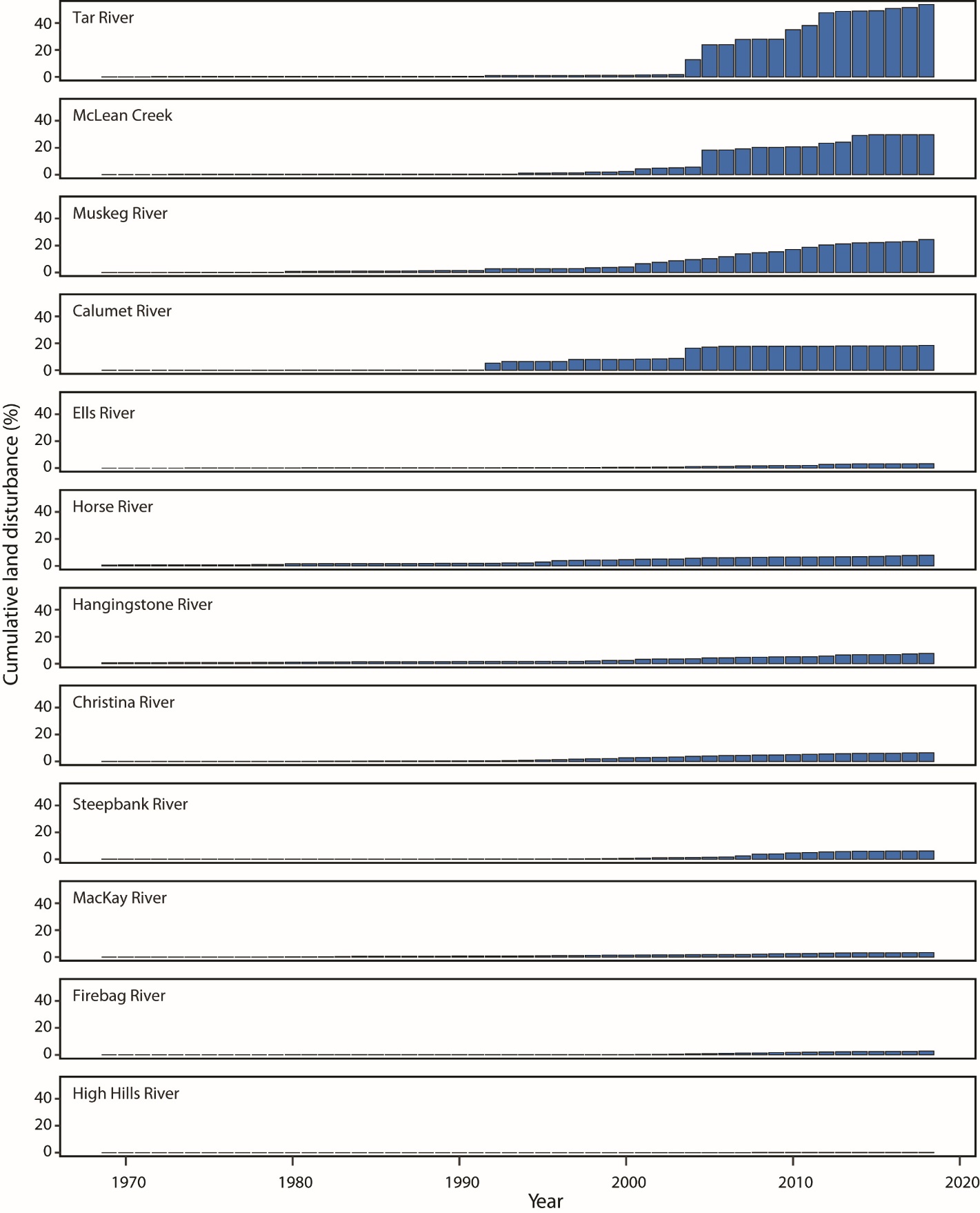


Supplemental Figure 12 Estimated cumulative landscape disturbance for each major watershed in the Athabasca and Cold Lake regions and ranked from top to bottom by disturbance greatest disturbance in 2018; calculated from ABMI 2018 Human Footprint data (<http://ftp.public.abmi.ca/GISData/HumanFootprint/2018/HFI_2018_v1.gdb.zip>).


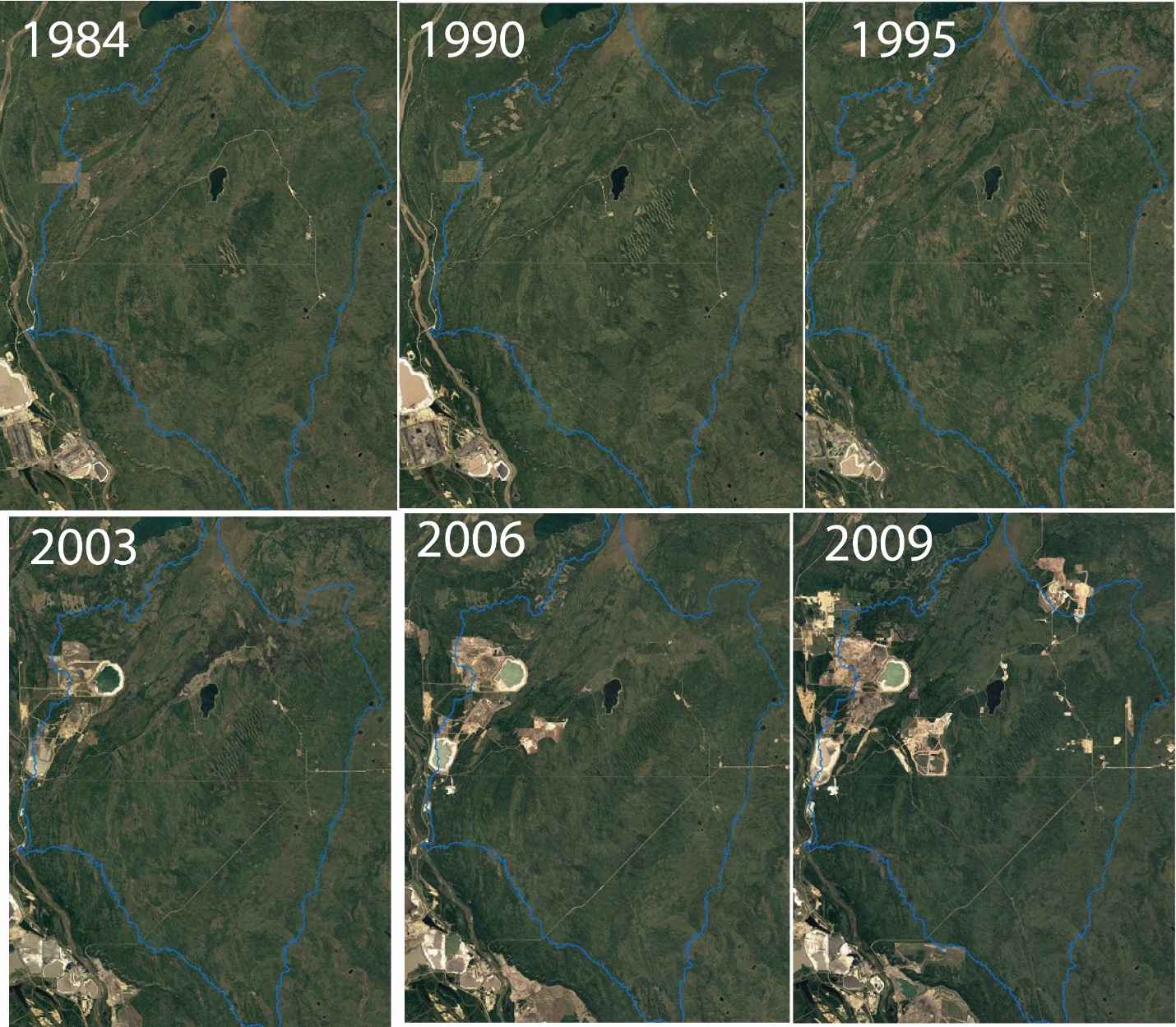


Supplemental Figure 13 Muskeg River satellite imagery over time including earliest imagery available through Google Earth (1984) and years of Fish Fence deployments (1995, 2003, 2006, and 2009); muskeg basin outlined in blue.


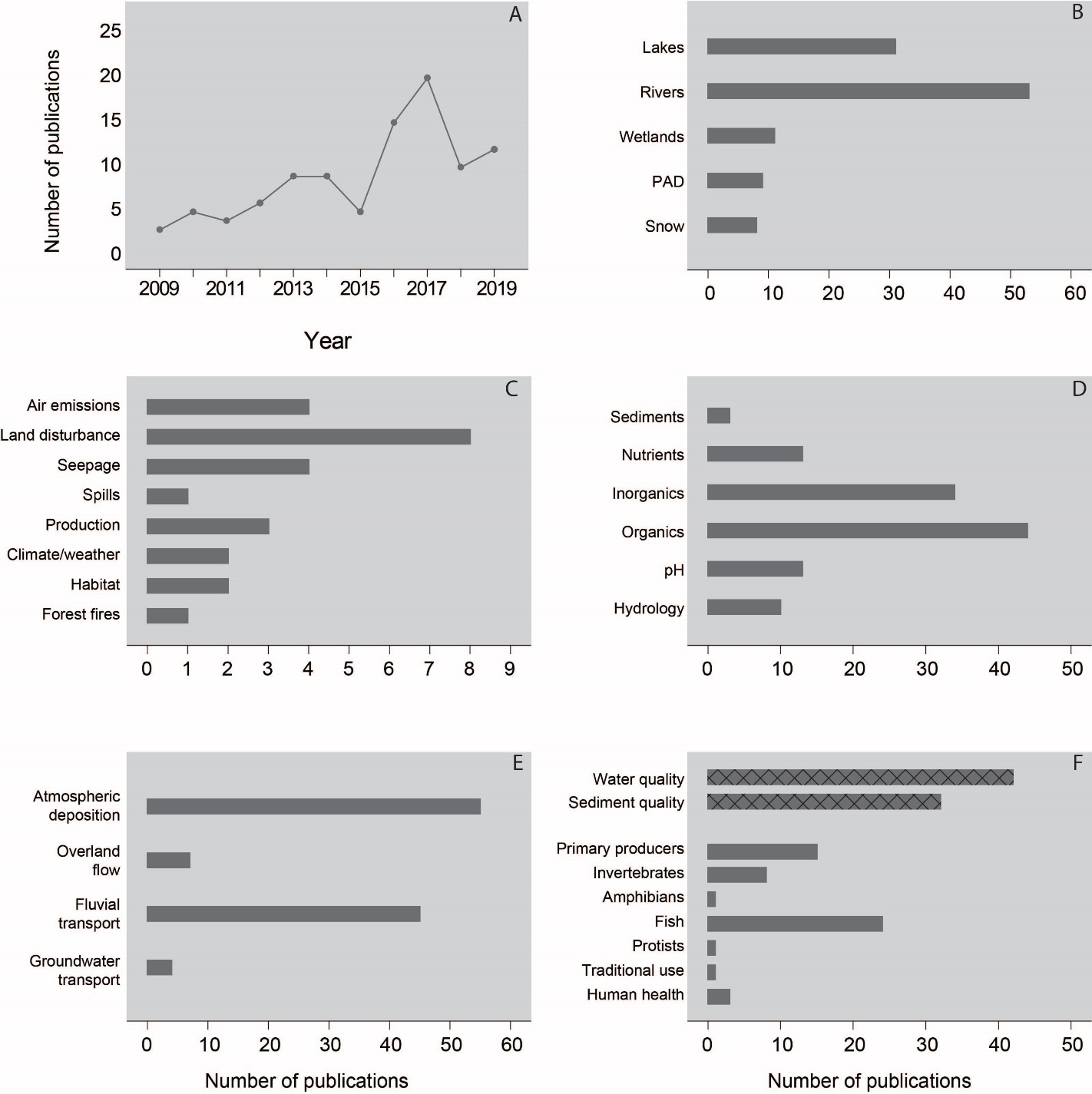


Supplemental Figure 14 Summary information for publications (123) discovered for inclusion in construction of conceptual model; (A) number of publications per year between 2009 and 2020; number of publications by habitat (B), pressures (C), stressors (D), pathways (E), and responses (F); hatched bars denote chemical responses in aquatic environments.
